# Angiopoietin-like 4 governs diurnal lipoprotein lipase activity in brown adipose tissue

**DOI:** 10.1016/j.molmet.2022.101497

**Published:** 2022-04-10

**Authors:** Robin van Eenige, Wietse In het Panhuis, Milena Schönke, Céline Jouffe, Thomas H. Devilee, Ricky Siebeler, Trea C.M. Streefland, Hetty C.M. Sips, Amanda C.M. Pronk, Ruben H.P. Vorderman, Hailiang Mei, Jan Bert van Klinken, Michel van Weeghel, Nina H. Uhlenhaut, Sander Kersten, Patrick C.N. Rensen, Sander Kooijman

**Affiliations:** 1 Division of Endocrinology, Department of Medicine, Leiden University Medical Center, Leiden, the Netherlands; 2 Einthoven Laboratory for Experimental Vascular Medicine, Leiden University Medical Center, Leiden, the Netherlands; 3 Institute for Diabetes and Endocrinology (IDE), Helmholtz Diabetes Center (HMGU) and German Center for Diabetes Research (DZD), Munich, Germany; 4 Sequencing Analysis Support Core, Department of Biomedical Data Sciences, Leiden University Medical Center, Leiden, the Netherlands; 5 Laboratory Genetic Metabolic Diseases, Amsterdam UMC, University of Amsterdam, Amsterdam Gastroenterology and Metabolism, Amsterdam Cardiovascular Sciences, Amsterdam, the Netherlands; 6 Core Facility Metabolomics, Amsterdam UMC, University of Amsterdam, Amsterdam, the Netherlands; 7 Department of Human Genetics, Leiden University Medical Center, Leiden, the Netherlands; 8 Metabolic Programming, Technical University of Munich School of Life Sciences, Freising, Germany; 9 Nutrition, Metabolism and Genomics Group, Division of Human Nutrition, Wageningen, the Netherlands

**Keywords:** Angiopoietin-like 4, Brown adipose tissue, Circadian/diurnal rhythms, Lipoprotein lipase, Peroxisome proliferator-activated receptor gamma, Transcriptomics

## Abstract

**Objective:**

Brown adipose tissue (BAT) burns fatty acids (FAs) to produce heat, and shows diurnal oscillation in glucose and triglyceride (TG)-derived FA-uptake, peaking around wakening. Here we aimed to gain insight in the diurnal regulation of metabolic BAT activity.

**Methods:**

RNA-sequencing, chromatin immunoprecipitation (ChIP)-sequencing, and lipidomics analyses were performed on BAT samples of wild type C57BL/6J mice collected at 3-hour intervals throughout the day. Knockout and overexpression models were used to study causal relationships in diurnal lipid handling by BAT.

**Results:**

We identified pronounced enrichment of oscillating genes involved in extracellular lipolysis in BAT, accompanied by oscillations of FA and monoacylglycerol content. This coincided with peak lipoprotein lipase (*Lpl*) expression, and was predicted to be driven by peroxisome proliferator-activated receptor gamma (PPARγ) activity. ChIP-sequencing for PPARγ confirmed oscillation in binding of PPARγ to *Lpl*. Of the known LPL-modulators, angiopoietin-like 4 (*Angptl4*) showed the largest diurnal amplitude opposite to *Lpl*, and both *Angptl4* knockout and overexpression attenuated oscillations of LPL activity and TG-derived FA-uptake by BAT.

**Conclusions:**

Our findings highlight involvement of PPARγ and a crucial role of ANGPTL4 in mediating the diurnal oscillation of TG-derived FA-uptake by BAT, and imply that time of day is essential when targeting LPL activity in BAT to improve metabolic health.

## Highlights

• The transcriptome and lipidome of brown fat show clusters with distinct circadian phases.
• The peak in metabolic brown fat activity is defined by activation of lipolytic processes.
• PPARγ shows oscillating binding to lipolytic genes and may drive diurnal brown fat activity.
• Genetic modulation of the lipoprotein lipase inhibitor *Angptl4* flattens rhythmic activity in brown fat.
• Time of day should be considered when studying the metabolic benefits of targeting brown fat.

## Graphical abstract

**Figure:**
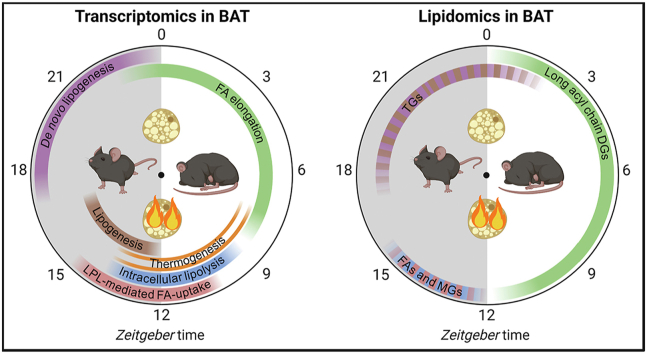

## Abbreviations

ABCD1: ATP binding cassette subfamily D member 1
ACACA: Acetyl-CoA carboxylase
ACLY: ATP citrate lyase
ACSL1: Acyl-CoA synthetase long chain family member 1
ANGPTL: Angiopoietin-like
APO: Apolipoprotein
BAT: Brown adipose tissue
CD36: Cluster of differentiation 36
CEBPA: CCAAT/enhancer-binding protein alpha
CEBPB: CCAAT/enhancer-binding protein beta
CES1D: Carboxylesterase 1 D
ChEA3: ChIP-X enrichment analysis version 3
CIDEC: Cell death inducing DFFA like effector C
CLOCK: Circadian locomotor output cycles kaput
DBP: Nuclear receptor subfamily 1 group D member
DG: Diacylglycerol
DGAT2: Diacylglycerol O-acyltransferase 2
ELOVL: Elongation of very long chain fatty acids protein
FA: Fatty acid
FASN: Fatty acid synthase
GAPDH: Glyceraldehyde 3-phosphate dehydrogenase
GCK: Glucokinase
GPAT4: Glycerol-3-phosphate acyltransferase 4
GPR: G-protein-coupled receptor
GPIHBP1: Glycosylphosphatidylinositol anchored high density lipoprotein binding protein 1
GTF2I: General transcription factor II-I
HACD4: 3-hydroxyacyl-CoA dehydratase 4
HDL: High-density lipoprotein
HRMS: High-resolution mass spectrometry
iBAT: Interscapular brown adipose tissue
KO: Knockout
LPL: Lipoprotein lipase
MG: Monoacylglycerol
MT-CYTB: Mitochondrial cytochrome b
MT-ND: NADH-ubiquinone oxidoreductase chain
NOCT: Nocturnin
PLIN4: Perilipin 4
PLTP: Phospholipid transfer protein
PNPLA2: Patatin like phospholipase domain containing 2
PPARγ: Peroxisome proliferator-activated receptor gamma
PUFA: Poly-unsaturated fatty acid
qPCR: Quantitative polymerase chain reaction
RPLP0: 60S acidic ribosomal protein P0
sBAT: Subscapular brown adipose tissue
SCD1: Stearoyl-CoA desaturase-1
SREBF1: Sterol regulatory element-binding transcription factor 1
TECR: Trans-2,3-enoyl-CoA reductase
TG: Triglyceride
Tg: Transgenic overexpression
TRL: Triglyceride-rich lipoprotein
UCP1: Uncoupling protein 1
UPLC: Ultra-performance liquid chromatography
WAT: White adipose tissue
WT: Wild type
ZT: *Zeitgeber* time

## 1 Introduction

White adipose tissue (WAT) is the most abundant type of adipose tissue that humans and other mammals have, which primarily stores energy from food. In contrast, the main function of brown adipose tissue (BAT) is to convert energy into heat, a process known as non-shivering thermogenesis. These two types are intertwined and highly related to one another as for example white adipocytes are capable of transdifferentiating into so-called beige adipocytes with brown-like characteristics. Brown and beige adipocytes produce heat via expression of uncoupling protein 1 (UCP1), which dissipates the mitochondrial proton motive force generated by fatty acid (FA) oxidation thereby releasing energy as heat [1]. Cold exposure is the main physiological activator of non-shivering thermogenesis and triggers the breakdown of intracellularly stored triglycerides (TGs) into FAs. To replenish intracellular lipid stores, BAT takes up glucose and FAs from circulating TG-rich lipoproteins (TRLs) [2]. Consequently, the uptake of nutrients by BAT can be used as a proxy for the presence and metabolic activity of this tissue [[3], [4], [5], [6]]. Importantly, because BAT has the capability of clearing large amounts of glucose and TG-derived FAs from the circulation, many favorable metabolic effects have been attributed to its thermogenic activity in mice, including the protection from atherosclerosis development [7]. Likewise, the presence of active BAT in humans has been associated with cardiometabolic health [8], highlighting its potential as therapeutic target.

We and others have shown that nutrient uptake by BAT is characterized by strong diurnal oscillations, with peak uptake of glucose and TG-derived FAs around wakening [[9], [10], [11], [12], [13], [14], [15]]. This diurnal pattern is most likely related to the daily need for non-shivering thermogenesis to maintain body temperature and/or facilitate the rise in body temperature prior to wakening. We hypothesized that insight in the mechanism driving oscillations of BAT activity may lead to the identification or optimization of strategies to promote thermogenesis, and may provide an explanation for the increased incidence of cardiometabolic diseases among shift workers [16]. Thus far, studies in rodents have revealed that, for example, the core-clock protein REV-ERBA suppresses *Ucp1* expression during the wakeful (active) phase [17]. Interestingly, cold exposure appears to add an extra dimension to the diurnal regulation of BAT as it suppresses *Nr1d1* expression (encoding REV-ERBA) to relieve the inhibition on *Ucp1* expression [17]. Similarly, Adlanmerini et al. [18] recently revealed that cold exposure introduces oscillation of genes involved in *de novo* lipogenesis with peak expression near the end of the feeding (active) phase of mice, which probably serves to provide BAT with extra fuel to maintain body temperature during the resting (inactive) phase and/or for combustion towards the start of the subsequent wakeful phase.

Here we aimed to gain further insight into the diurnal regulation of metabolic BAT activity. To this end we used an unbiased RNA sequencing and lipidomics approach in BAT of mice, and identified angiopoietin-like (*Angptl*)*4* as critical mediator in the diurnal regulation of lipoprotein lipase (LPL) activity, thereby governing the diurnal regulation of TG-derived FA-uptake by BAT.

## 2 Results & discussion

### 2.1 The transcriptome of murine brown adipose tissue consists of four clusters with distinct oscillating expression phases

**Figure 1:**
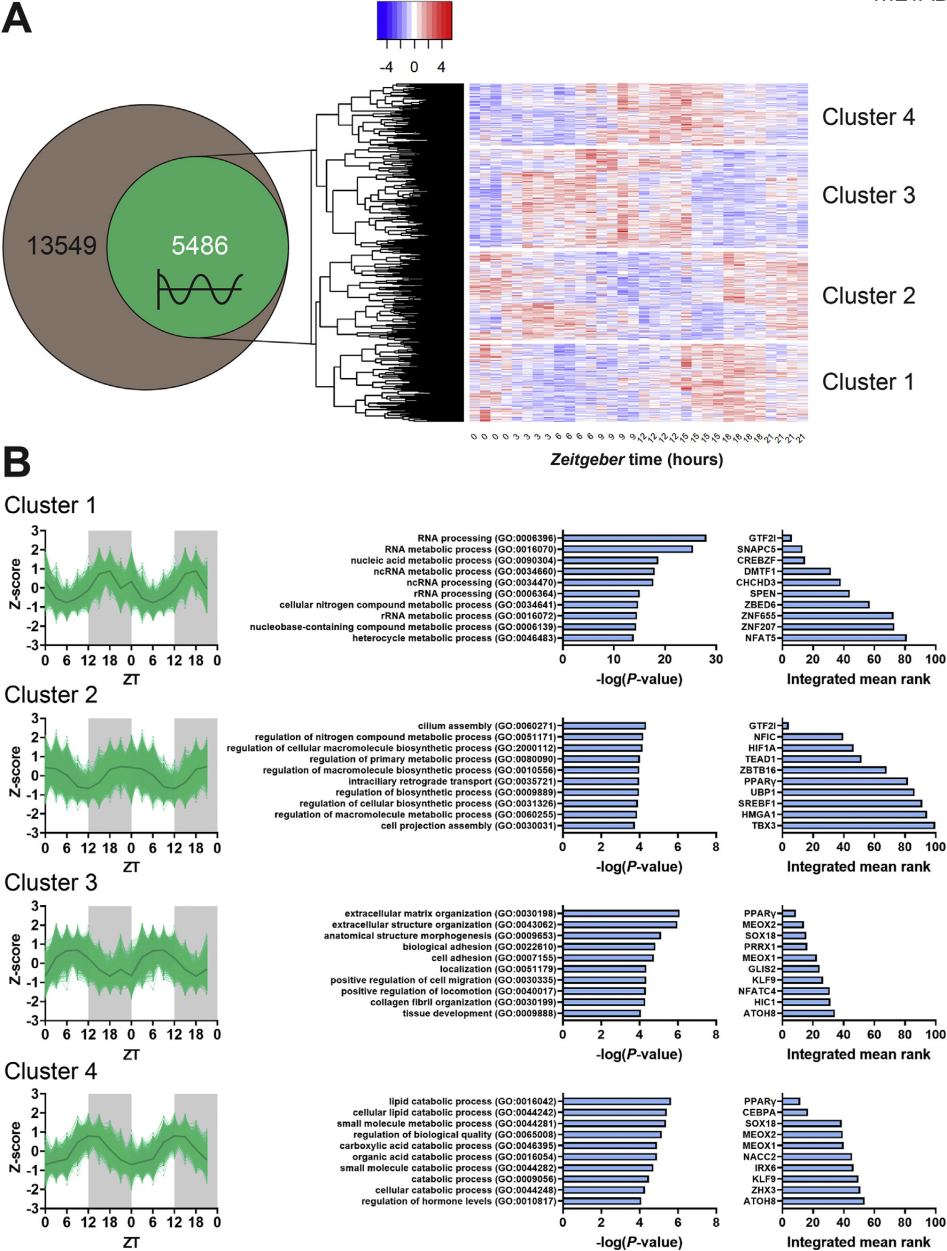
**The transcriptome of murine brown adipose tissue consists of four clusters with distinct oscillating expression phases**. Interscapular brown adipose tissue samples were collected from chow-fed male C57BL/6J mice at 3-hour intervals throughout a 24-hour period to produce eight time points in total (*n* = 4 per time point), which were used to perform RNA-sequencing. Oscillation was assessed by JTK, and **(A)** oscillating (*P* < 0.05) genes as a subset of all expressed genes were visualized in a Venn diagram. Hierarchical clustering of standardized residuals (Z-scores) of all oscillating genes was visualized in a heat map and **(B)** expression of genes within the four major clusters was double plotted for visual purposes. Functional enrichment by gene ontology was performed on each cluster, as well as enrichment by transcription factor of which top 10 hits are depicted. ATOH8, atonal BHLH transcription factor 8; CEBPA, CCAAT enhancer binding protein alpha; CHCHD3, coiled-coil-helix-coiled-coil-helix domain containing 3; CREBZF, CREB/ATF BZIP transcription factor; DMTF1, cyclin D binding MYB like transcription factor 1; GLIS2, GLIS family zinc finger 2; GTF2I, general transcription factor IIi; HIC1, HIC ZBTB transcriptional repressor 1; HIF1A, hypoxia inducible factor 1 subunit alpha; HMGA1, high mobility group AT-hook 1; IRX6, iroquois homeobox 6; KLF9, Kruppel like factor 6; MEOX1, mesenchyme homeobox 1; MEOX2, mesenchyme homeobox 2; NACC2, NACC family member 2; NFAT5, nuclear factor of activated T cells 5; NFATC4, nuclear factor of activated T cells 4; NFIC, nuclear factor I C; PPARγ, peroxisome proliferator activated receptor gamma; PRRX1, paired related homeobox 1; SNAPC5, small nuclear RNA activating complex polypeptide 5; SOX18, SRY-box transcription factor 18; SPEN, spen family transcriptional repressor; SREBF1, sterol regulatory element binding transcription factor 1; TBX3, T-box transcription factor 3; TEAD1, TEA domain transcription factor 1; UBP1, upstream binding protein 1; ZBED6, zinc finger bed-type containing 6; ZBTB16, zinc finger and BTB domain containing 16; ZHX3, zinc fingers and homeoboxes 3; ZNF, zinc finger protein.

RNA-sequencing was performed on interscapular BAT (iBAT) samples collected at 3-hour intervals throughout a 24-hour period from chow-fed male C57BL/6J mice exposed to mild cold (22 °C, which is approximately 8 °C below the thermoneutral zone [19]). All mice were entrained to a 12h:12h light:dark cycle, and therefore time is denoted as *Zeitgeber* Time (ZT) where ZT0 indicates the onset of the light (inactive) phase. Oscillation of transcripts was assessed by JTK cycle analysis, which provided the phase, amplitude, and significance values for each transcript and showed that in total, 5,486 genes (40.5% of all expressed genes; Figure 1A) were oscillating (*P* < 0.05) (full details are provided in supplementary file: ‘RNA-sequencing’). Within this selection of oscillating genes, hierarchical clustering of standardized residuals identified four gene clusters with distinct expression phases (Figure 1A). Additional horizontal clustering showed that samples collected at the same or sequential time points clustered together, highlighting their close similarities (Suppl Fig S1). On the four identified gene clusters, we carried out gene ontology and transcription factor enrichment analyses.

Cluster 1 (consisting of 1,314 genes) showed peak expression around ZT18 (Figure 1B) and was enriched in genes involved in posttranscriptional RNA processing (top hit GO:0006396; *P* = 7.77 · 10^−29^) (Figure 1B). These pathways are under direct control of the cellular clock machinery and contribute to the circadian regulation of gene expression in many tissues [20].

Cluster 2 (consisting of 1,499 genes) peaked around the onset of the light phase at ZT0 (Figure 1B), and was enriched in genes involved in metabolic and biosynthetic processes, with the top hit being cilium assembly (GO:0060271; *P* = 4.56 · 10^−7^) (Figure 1B). This is of interest because primary cilia have a pivotal role in signaling pathways, including the transduction of signals that promote adipogenesis. In addition, many G-protein-coupled receptors (GPR) have been found to be selectively targeted by cilia. Whether this is true for the GPRs implicated in BAT functioning such as the β-adrenergic receptors [21,22], GPR120 [23] and GPR3 [24] is unknown, but represents an interesting topic for further studies.

Transcription factor enrichment analysis identified general transcription factor II-I (GTF2I) as a potential mediator of the gene expression in clusters 1 and 2 (Figure 1B). GTF2I is involved in various aspects of general cell physiology and is known to interact with the circadian locomotor output cycles kaput (CLOCK) [25]. Within cluster 2, also oscillating genes involved in *de novo* lipogenesis were identified (Suppl Fig S2) in line with recent findings [18], including ATP citrate lyase (*Acly*) and acetyl-CoA carboxylase (*Acaca*). Glucokinase (*Gck*), another gene involved in *de novo* lipogenesis, displayed oscillation with a slightly earlier peak expression at ZT18 corresponding to cluster 1. Despite the sub-thermoneutral housing temperature of 22 °C, no oscillations were identified for FA synthase (*Fasn*)*,* stearoyl-CoA desaturase-1 (*Scd1*), and elongation of very long chain FAs protein (*Elovl*)*3* (Suppl Fig S2). This indicates that the dependency on *de novo* lipogenesis may be determined by the degree of cold exposure, as severe cold stress induces oscillations of more *de novo* lipogenic genes [18] than we observed at mild cold stress.

Cluster 3 (consisting of 1,660 genes) had a peak expression around ZT8 (Figure 1B), and was enriched in genes involved in among others cellular organization, adhesion, and localization, with extracellular matrix organization as top hit (GO:0030198; *P* = 7.92 · 10^−7^; Figure 1B). As yet, little is known about the interaction between circadian clocks and the cellular microenvironment in BAT or other tissues, which thus warrants further exploration.

Lastly, cluster 4 (consisting of 1013 genes) was identified with peak expression around ZT14 (Figure 1B), coinciding with the previously reported peak in metabolic BAT activity as defined by TG-derived FA-uptake [13]. Correspondingly, this cluster showed enrichment in catabolic processes, with the top hit being lipid catabolic process (GO:0016042; *P* = 2.31·10^−6^) (Figure 1B).

Transcription factor enrichment analysis revealed peroxisome proliferator-activated receptor gamma (PPARγ) as the top hit for both cluster 3 and 4. PPARγ senses the energy status of the cell to regulate lipid uptake and storage, and in fact has often been described as an important circadian transcription factor in adipose tissue [26,27]. Its expression is regulated by CCAAT/enhancer-binding protein alpha (CEBPA), which we identified as first hit following PPARγ in cluster 4 (Figure 1B), and its activity is enhanced upon interaction with the circadian protein nocturnin (NOCT) [26,28]. In our dataset, *Cebpa* expression by itself was found oscillating within cluster 3, while expression of *Pparg* itself and *Noct* were not oscillating (data not shown), likely because PPARγ is broadly regulated at the post-transcriptional level [26]. Strikingly, transcription factor enrichment analysis on the top 100 genes with the largest absolute oscillation amplitude among all oscillating genes also identified PPARγ as the top hit (Suppl Fig S3). CEBPA, and CCAAT/enhancer-binding protein beta (CEBPB), both regulators of PPARγ, also showed up in the top 10 of this transcription factor enrichment analysis, as well as sterol regulatory element binding transcription factor 1 (SREBF1) (also known as SREBP1), which is required for *de novo* lipogenesis [29] and is regulated by PPARγ [30]. Collectively, these data predict a central role for PPARγ in driving transcriptional oscillation within BAT, likely mediated by post-transcriptional regulation.

To obtain further insight in the processes that take place during the time that BAT is metabolically most active (*i.e.*, around ZT12), relative amplitudes of individual genes were compared within clusters 3 and 4 (Suppl Table S1). We identified nuclear receptor subfamily 1 group D member 1 (*Nr1d1*) and D-box binding protein (*Dbp*), both involved in the core clock machinery, as the genes with largest relative amplitude in cluster 3 and 4, respectively. The genes that follow in cluster 3 are involved in a broad variety of cellular processes including cytokine binding and amino acid metabolism, while the three genes that follow in cluster 4 are all involved in the cellular core clock machinery.

Although of interest, these data do not explain the diurnal metabolic activity in BAT, and we therefore proceeded with comparing absolute amplitudes of individual genes (Suppl Table S1). NADH-ubiquinone oxidoreductase chain 1 (*mt-nd1*), a subunit of NADH dehydrogenase critical for the electron transport chain, was identified as the gene with the highest diurnal amplitude within the third cluster, with estimated peak expression near ZT8. The four genes that follow within this cluster encode for mitochondrial complexes and *Ucp1*, suggesting that large diurnal changes in mitochondrial dynamics are required for the flexible regulation of FA combustion [[31], [32], [33]].

Interestingly, we identified a large absolute diurnal amplitude for *Lpl*, encoding for the protein responsible for liberating FAs from TRLs [2], in cluster 4 with estimated peak expression at ZT12. Other genes with large amplitudes in clusters 3 and 4 were identified as being involved in lipid storage (*i.e.* glycerol-3-phosphate acyltransferase 4 (*Gpat4*), diacylglycerol O-acyltransferase 2 (*Dgat2*), cell death inducing DFFA like effector C (*Cidec*), and Perilipin 4 (*Plin4*)) and intracellular lipolysis (*i.e.* acyl-CoA synthetase long chain family member 1 (*Acsl1*), patatin like phospholipase domain containing 2 (*Pnpla2*), and carboxylesterase 1 D (*Ces1d*)).

**Figure 2:**
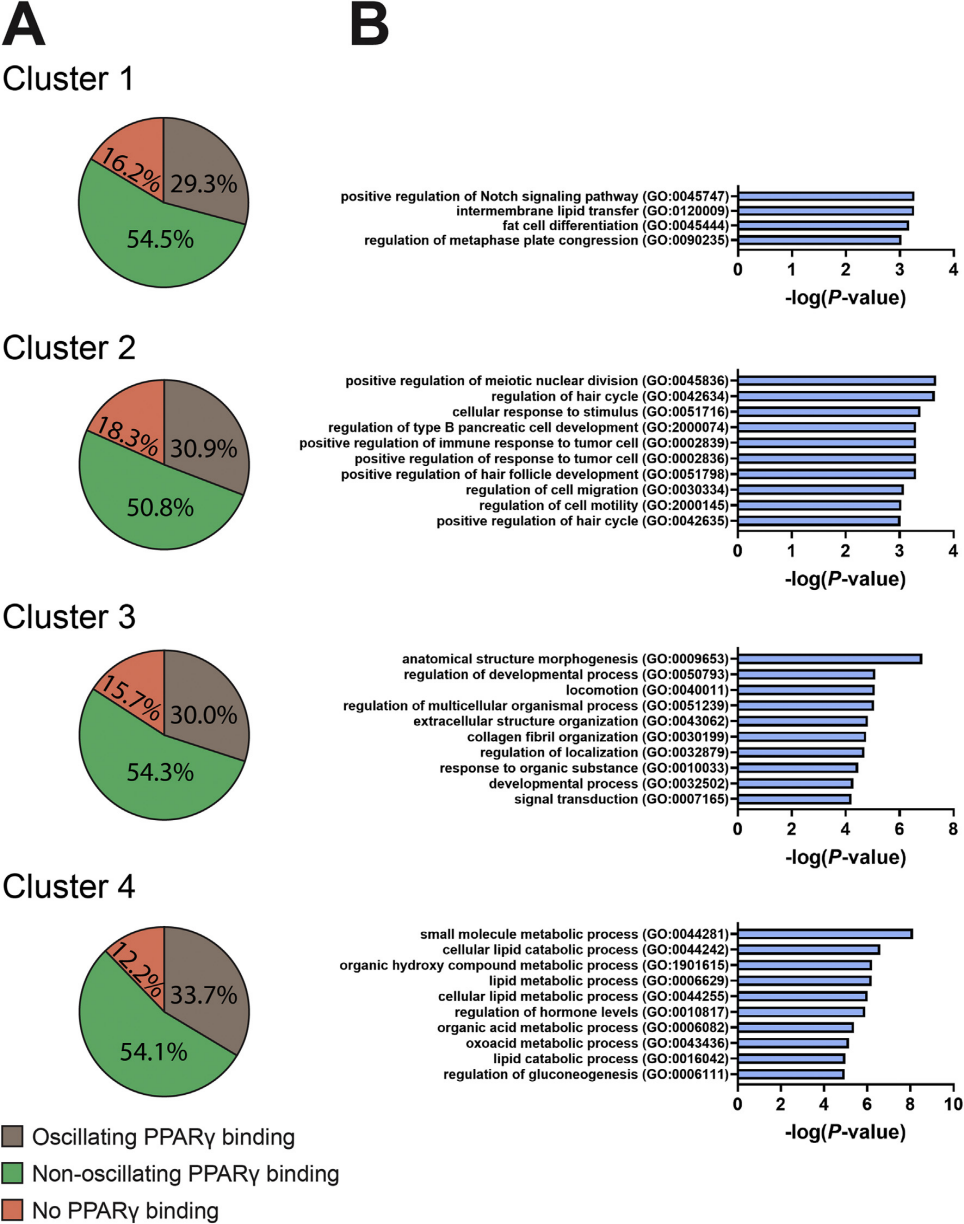
**Enrichment for (lipid) catabolic processes in oscillating genes with oscillating PPARγ binding**. Interscapular brown adipose tissue samples were collected from chow-fed male C57BL/6J mice at 3-hour intervals throughout a 24-hour period to produce eight time points in total (*n* = 8 per time point). On pooled samples, chromatin immunoprecipitation (ChIP)-sequencing was performed for PPARγ, and oscillation of peaks was assessed by JTK. Peaks were annotated, and within each of the four gene clusters with distinct expression phases as identified by RNA-sequencing (Figure 1), **(A)** proportions of genes with oscillating (*P* < 0.05) PPARγ binding, non-oscillating PPARγ binding, or no PPARγ binding were visualized in Pie charts. **(B)** Functional enrichment by gene ontology was performed on the genes with oscillating PPARγ binding, and top 10 hits are depicted. For cluster 1, all four hits are depicted.

To substantiate the idea that PPARγ is involved in the regulation of diurnal gene expression in clusters 3 and 4, we performed chromatin immunoprecipitation (ChIP)-sequencing for PPARγ on pooled iBAT samples. Out of 66,066 peaks (*i.e.* PPARγ binding sites), 8,153 demonstrated diurnal oscillation as determined by JTK-cycle analysis (full details are provided in supplementary file: ‘ChIP-sequencing’). Peaks were annotated to genes associated with the nearest transcriptional start site, and these data were used to identify the genes with oscillating PPARγ binding and oscillating gene expression (Figure 2A; Suppl Fig S4). Strikingly, within cluster 4, genes with oscillating PPARγ binding showed gene ontology enrichment in (lipid) catabolic processes (Figure 2B), while genes with non-oscillating PPARγ binding did not show such enrichment (Suppl Fig S5). These data suggest a role of PPARγ in regulating transcription of genes involved in intracellular and extracellular lipolysis when BAT is metabolically most active. This notion is further strengthened by strong oscillations in PPARγ binding to sites annotated to abovementioned genes with largest diurnal amplitude (Suppl Table S1) that are involved in lipid storage and intracellular lipolysis (*i.e. Acsl1, Ces1d*, *Dgat2*, *Gpat4*, *Plin4* and *Pnpla2*), with estimated peaks in PPARγ binding on average 2 h prior to estimated peak in gene expression (Suppl Fig S6). Notably, six oscillating binding sites that were annotated to *Lpl* could be identified with estimated peak in PPARγ binding at ZT6-7.5 (Suppl Fig S7).

From these results we interpret that intracellular lipolysis at the end of the light (inactive) phase serves to supply BAT with fuel for thermogenesis. This seems to be followed by storage of FA taken up from the circulation after hydrolysis of TRLs by LPL and subsequent lipogenesis around the onset of the dark (active) phase, likely in order to replenish intracellular lipid stores. PPARγ likely mediates as a driving factor in the transcriptional control of genes involved in the uptake, storage, and intracellular lipolysis of lipids.

### 2.2 Oscillations of genes involved in intracellular and extracellular lipolysis are in synchrony with oscillations of fatty acids and monoacylglycerols

**Figure 3:**
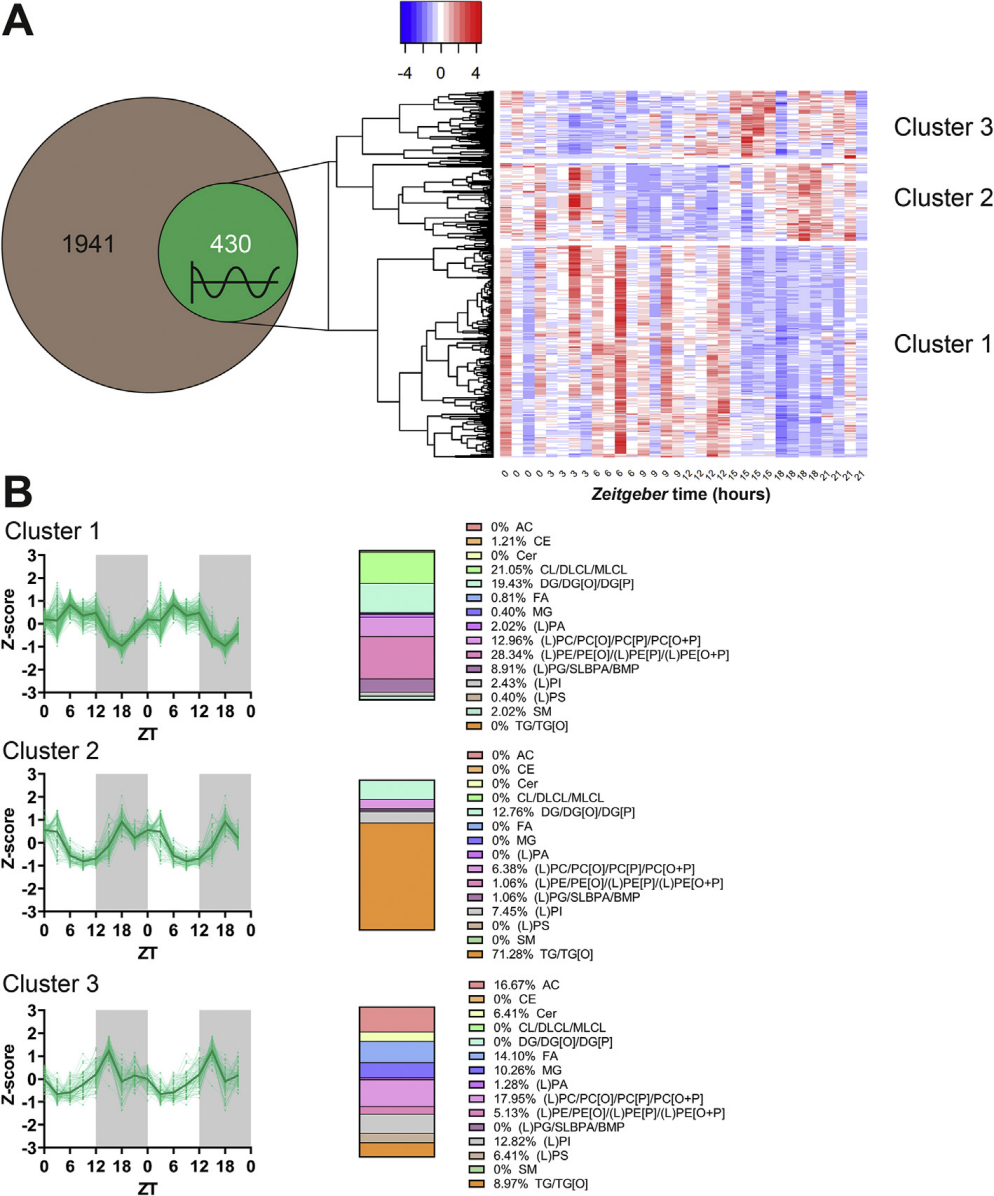
**The lipidome of murine brown adipose tissue consists of three clusters with distinct oscillating phases**. Interscapular brown adipose tissue samples were collected from chow-fed male C57BL/6J mice at 3-hour intervals throughout a 24-hour period to produce eight time points in total (*n* = 4 per time point), which were used to perform ultra-performance liquid chromatography (UPLC)-high-resolution mass spectrometry (HRMS)-based lipidomics. Oscillation was assessed by JTK, and **(A)** oscillating (*P* < 0.05) lipid species as a subset of all measured lipids were visualized in a Venn diagram. Hierarchical clustering of standardized residuals (Z-scores) of oscillating lipid species was visualized in a heat map and **(B)** levels within the three major clusters were double plotted for visual purposes. Within each cluster, the contribution of each lipid category to its composition is presented. AC, acylcarnitine; CE, cholesteryl ester; Cer, ceramide; CL, cardiolipin; DG, diacylglycerol; DLCL, dilysocardiolipin; FA, fatty acid; LPA, lysophosphatic acid; LPE, lysophosphatidylethanolamine; MG, monoacylglycerol; LPG, lysophosphatidylglycerol; LPI, lysophosphatidylinositol; LPS, lysophosphatidylserine; MLCL, monolysocardiolipin; PA, phosphatic acid; PC, phosphatidylcholine; PE, phosphatidylethanolamine; PG, phosphatidylglycerols; PI, phosphatidylinositols; PS, phosphatidylserine; SM, sphingomyelin; TG, triglyceride. [O] and [P] refer to the presence of an alkyl ether or 1Z-alkenyl ether substituent, respectively.

To investigate how diurnal transcriptional oscillations relate to diurnal changes in lipid content, we next carried out ultra-performance liquid chromatography (UPLC)-high-resolution mass spectrometry (HRMS)-based lipidomics on the same iBAT samples. Out of 1,941 measured lipid species, 430 demonstrated diurnal oscillation as analyzed by JTK (Figure 3A) (full details are provided in supplementary file: ‘Lipidomics’). For 11 of those lipid species, concentrations in some samples were below the detection limit and were therefore excluded from further analysis. Standardized residuals of oscillating lipids were subjected to hierarchical clustering, which identified three clusters (Figure 3A–B), with estimated peaks at ZT7 (cluster 1; 247 lipids), ZT21 (cluster 2; 78 lipids), and ZT16 (cluster 3; 94 lipids) (Figure 3B). A heatmap with additional horizontal (*i.e.* sample) clustering is presented in Suppl Fig S8. Cluster 1 (peak at ZT7) contained 48 of the 60 oscillating diacylglycerols (DGs), of which 39 had at least one long acyl chain (≥C18:x), reflecting ongoing FA elongation during this period likely serving to store energy for efficient combustion at the start of the next wakeful phase. Correspondingly, of the six oscillating genes involved in FA elongation, ATP binding cassette subfamily D member 1 (*Abcd1*), *Elovl5*, 3-hydroxyacyl-CoA dehydratase 4 (*Hacd4*), and trans-2,3-enoyl-CoA reductase (*Tecr*) showed peak expression throughout the light phase (Suppl Fig S9).

Cluster 2 (peak ZT21) contained the remaining oscillating DGs, characterized by primarily short or medium acyl chains (<C18:x). In addition, this cluster contained 67 of the 74 oscillating TGs (Figure 3B), probably as the result of food intake, *de novo* lipogenesis, and metabolically inactive BAT.

Cluster 3 (peak ZT16) contained 11 of the 13 oscillating FAs and eight of the nine oscillating monoacylglycerols (MGs; Figure 3B). These data are in concordance with our previous observations in young mice [34], and consistent with a peak in lipolytic activity at the onset of the dark (active) phase as indicated by the RNA-sequencing data and previous functional data on TG-derived FA-uptake by BAT [13], as both intracellular and extracellular lipolysis yield MGs and FAs. Of note, five of the identified FAs within cluster 3 are poly-unsaturated FAs (PUFAs), which are known ligands for PPARγ [35] and UCP1 [36] and therefore likely contribute to thermogenic activation of the tissue followed by the uptake and storage of TG-derived FA from the circulation.

### 2.3 The lipoprotein lipase pathway follows diurnal oscillations with a peak at the onset of the active phase

**Figure 4:**
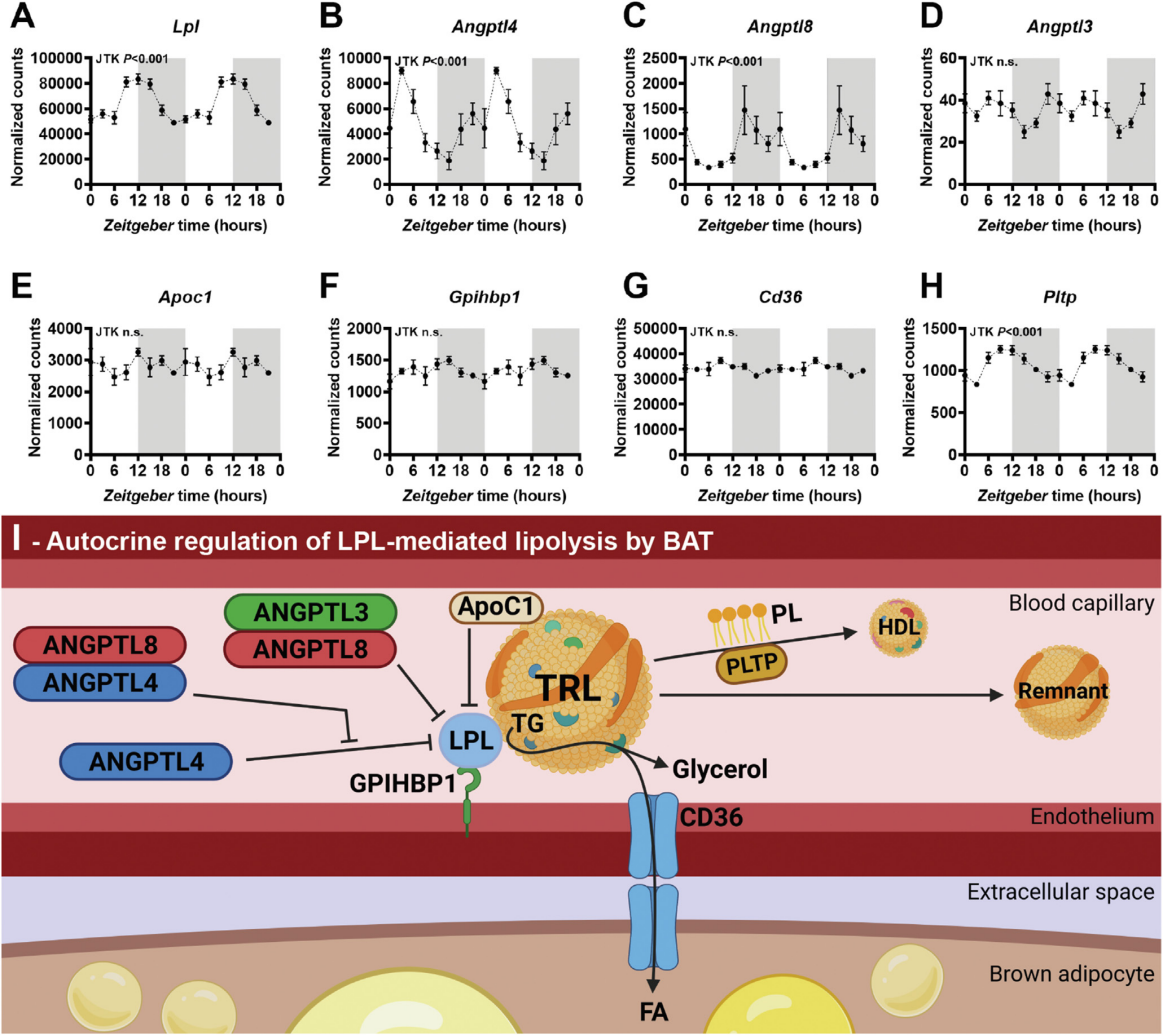
**The lipoprotein lipase pathway follows diurnal oscillations that peak at the onset of the active phase.** Interscapular brown adipose tissue (BAT) samples were collected from chow-fed male C57BL/6J mice at 3-hour intervals throughout a 24-hour period to produce eight time points in total (*n* = 4 per time point), which were used to perform RNA-sequencing. Oscillation was assessed by JTK, and normalized counts of **(A)** lipoprotein lipase (*Lpl*), **(B)** angiopoietin-like (*Angptl*)*4*, **(C)***Angptl8*, **(D)***Angptl3,***(E)** apolipoprotein C1 (*Apoc1*), **(F)** glycosylphosphatidylinositol anchored high density lipoprotein binding protein 1 (*Gpihbp1*), **(G)** cluster of differentiation 36 (*Cd36*), and **(H)** phospholipid transfer protein (*Pltp*) were double plotted. **(I)** A schematic overview of LPL-mediated triglyceride (TG)-rich lipoprotein (TRL) hydrolysis and the autocrine regulation hereof by BAT. FA, fatty acid; HDL, high-density lipoprotein; PL, phospholipid.

We observed peak expression of genes involved in lipid catabolic processes around ZT12 (onset of the dark phase) and identified *Lpl* as the gene with the largest amplitude within this cluster, highlighting its relevance in the diurnal oscillation of TG-derived FA-uptake by BAT [13]. To delineate which components of this TRL-processing pathway are oscillating, we next characterized the diurnal expression of genes involved in the LPL-mediated lipolytic processing of TRLs, as well as cellular uptake and transport of TG-derived FAs. Hereto, we visualized the diurnal oscillations of a manual selection of genes related to LPL-mediated TRL processing, including the LPL-regulators *Angptl3*, *Angptl4*, and *Angptl8*, apolipoprotein (*Apo*)*c1*, *Apoc2*, *Apoc3*, *Apoa5* [37], and related genes including glycosylphosphatidylinositol anchored high density lipoprotein binding protein 1 (*Gpihbp1*), FA transporter cluster of differentiation 36 (*Cd36*), and phospholipid transfer protein (*Pltp*). Strikingly, besides the strong oscillation of *Lpl* expression (Figure 4A), expression of the LPL-inhibitors [38,39] *Angptl4* and *Angptl8* was found to oscillate with high amplitude, while expression of *Angptl3* was low and not oscillating (Figure 4B–D). In particular, the expression of *Angptl4* showed a 5-fold difference between highest (ZT3) and lowest (ZT15) measured levels opposing the oscillation of *Lpl* expression (Figure 4B). It was previously reported that in WAT, oscillations of *Angptl4* expression follow the pattern in BAT albeit with a 5-hour delay [40], illustrating tissue-specific regulation and a possible prioritization of fuel utilization by BAT prior to wakening [40]. ANGPTL8 forms a complex with hepatic-derived ANGPTL3 to strengthen its inhibition of LPL, as well as with ANGPTL4 to counteract its inhibitory effect on LPL [41], therefore the opposing oscillation of *Angptl8* may amplify ANGPTL4 activity at its peak and further inhibit it at its nadir. Importantly, ANGPTLs modulate LPL on protein but not mRNA level, instead oscillations of *Lpl* gene expression may be driven by PPARγ as described above. Additionally, *Lpl* and *Angptl4* may be driven by daily fluctuations in insulin [1,42]. In contrast to ANGPTLs, apolipoproteins known to modulate LPL activity were either not oscillating (*e.g. Apoc1*; Figure 4E) or not detected in BAT (*e.g. Apoc2*, *Apoc3*, and *Apoa5*; data not shown). Similarly, gene expression of *Gpihbp1*, which protects LPL from catalysis by ANGPTLs, and the FA transporter *Cd36* showed no oscillation (Figure 4F–G). Interestingly, we identified oscillation in the expression of *Pltp* (Figure 4H). This protein contributes to the transfer of phospholipids from TRLs to high-density lipoprotein (HDL) following liberation during LPL-mediated lipolysis, thereby facilitating the transport of TRL-remnant-derived cholesterol to the liver, as part of reverse cholesterol transport [43]. A schematic overview of LPL-mediated lipolysis and the autocrine regulation hereof by BAT via these genes and corresponding proteins is depicted in Figure 4I.

Taken together, of the known LPL modulators expressed in BAT, *Angptl4* showed the largest diurnal amplitude and opposite to *Lpl*. Accordingly, we hypothesized that ANGPTL4 is the main regulator of the diurnal variation in LPL activity within BAT.

### 2.4 Angiopoietin-like 4 modulation flattens oscillation of lipoprotein lipase activity and triglyceride-derived fatty acid uptake by brown adipose tissue

**Figure 5:**
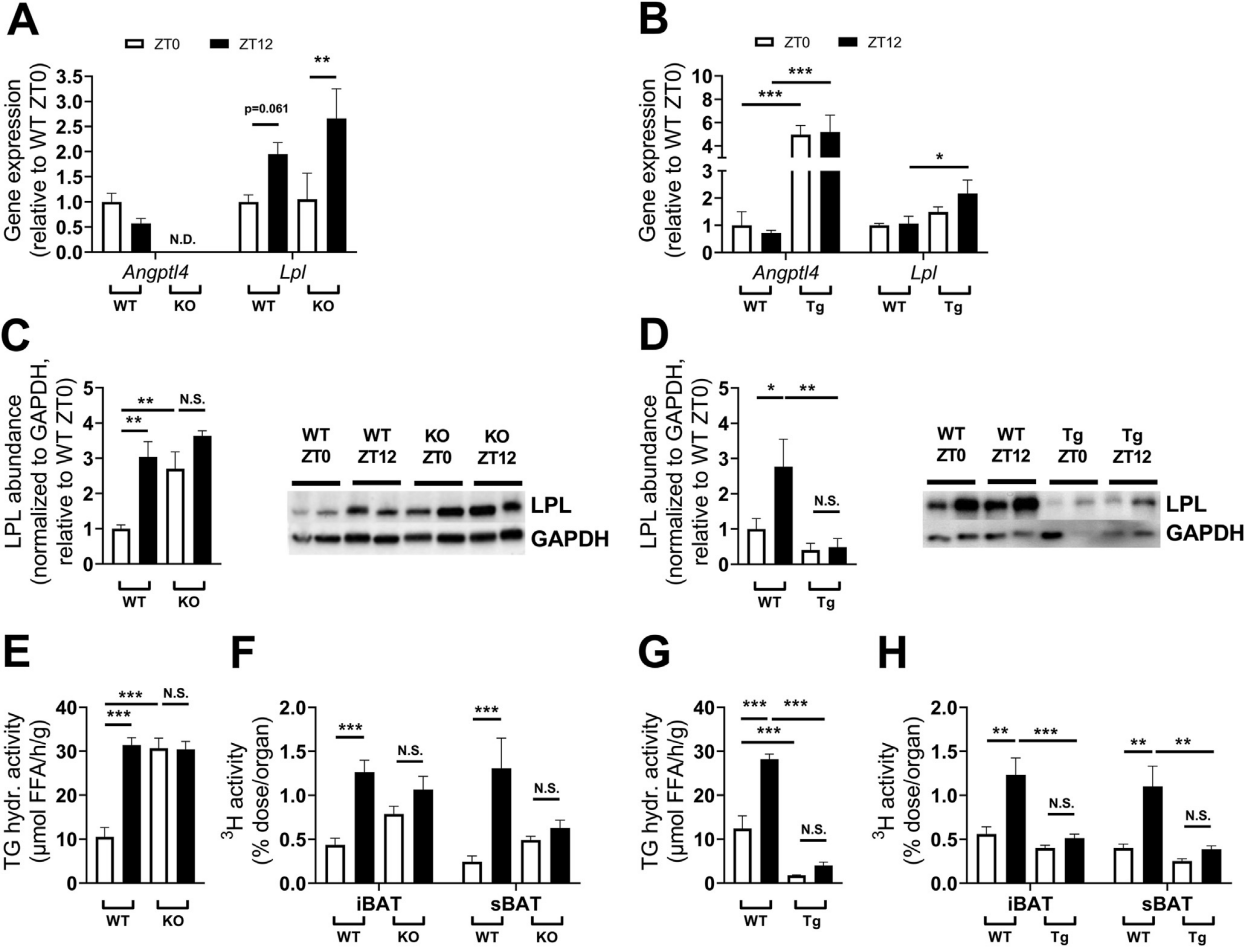
**Angiopoietin-like 4 modulation flattens oscillation of lipoprotein lipase activity and triglyceride-derived fatty acid uptake by brown adipose tissue.** Male wild type (WT), *angiopoietin-like 4* (*Angptl4*) knockout (KO) and *Angptl4* transgenic overexpression (Tg) mice were examined at the onset of the light (inactive) phase (*i.e. Zeitgeber* time (ZT)0) and dark (active) phase (*i.e.* ZT12). Gene expression of *Angptl4* and lipoprotein lipase (*Lpl*) was measured in subscapular brown adipose tissue (sBAT) of **(A)***Angptl4* KO and **(B)***Angptl4* Tg mice by quantitative polymerase chain reaction. Protein abundance of LPL was measured in interscapular (i)BAT of **(C)***Angptl4* KO and **(D)** Tg mice by Western blot, normalized to glyceraldehyde-3-phosphate dehydrogenase (GAPDH) levels. Representative images are shown. *In vitro* lipoprotein lipase (LPL) activity was measured in iBAT of **(E)***Angptl4* KO and **(G)***Angptl4* Tg mice and expressed as triglyceride (TG) hydrolyzing (hydr.) activity in μmol free fatty acids (FFA) per hour (h) per gram (g) tissue. Prior to killing, **(F)***Angptl4* KO and **(H)***Angptl4* Tg mice were injected with TG-rich lipoprotein-like particles labeled with glycerol tri[^3^H]oleate to assess fatty acid uptake by BAT. Data are presented as means ± SEM (*n* = 6–9 per group per time point). ∗Statistically significant compared to mice of equal genotype at a different timepoint or between groups as indicated by bars. ∗*P* < 0.05; ∗∗*P* < 0.01; ∗∗∗*P* < 0.001; N.D., not detectable. N.S., not significant.

To delineate the role of *Angptl4* in the diurnal regulation of LPL activity in BAT, we utilized whole-body *Angptl4* knockout (KO) and transgenic overexpression (Tg) mice, which we compared to wild type (WT) mice at ZT0 (corresponding to the nadir in *Lpl* expression) and ZT12 (corresponding to the peak in *Lpl* expression). Knockout (Figure 5A) or overexpression (Figure 5B) of *Angptl4* was confirmed by quantitative polymerase chain reaction (qPCR). Diurnal gene expression of *Lpl* was unaltered in *Angptl4* KO mice (Figure 5A), which is in line with the observation that ANGPTL4 inhibits LPL primarily at the protein rather than mRNA level [42]. On the other hand, gene expression of *Lpl* was increased in *Angptl4* Tg mice at ZT12 (Figure 5B), which might be a compensatory mechanism for reduced TG-derived FA-uptake due to highly suppressed LPL activity. Protein abundance of LPL was 3-fold higher at ZT12 compared to ZT0 in WT mice, in line with what was previously reported [13] (Figure 5C–D). In *Angptl4* KO mice and *Angptl4* Tg mice, LPL protein levels were not oscillating and equal to the abundance in WT mice at ZT12 and ZT0, respectively (Figure 5C–D), indicating that LPL is fully suppressed at ZT0 and fully activated at ZT12 in WT mice. The TG-hydrolase activity of iBAT-derived extracellular LPL was assessed *in vitro* (Figure 5E,G) and matched the abundance of LPL (Figure 5C–D). To determine the functional consequences on the diurnal oscillation of TG-derived FA-uptake by BAT, *Angptl4* KO and *Angptl4* Tg mice were injected with TRL-like particles containing glycerol tri[^3^H]oleate. In line with LPL abundance and activity, *Angptl4* KO mice showed constitutively increased [^3^H]oleate uptake by BAT (Figure 5F), and *Angptl4* Tg mice constitutively reduced [^3^H]oleate uptake by BAT (Figure 5H) compared to WT mice. Although generally regulated in the same direction, the abundance and TG-hydrolase activity of LPL *in vitro* at ZT0 did not translate one-to-one to TG-derived FA-uptake by BAT, indicating that other oscillating factors, such as ANGPTL8, may further finetune TG-derived FA-uptake *in vivo*.

## 3 Conclusions & perspective

**Figure 6:**
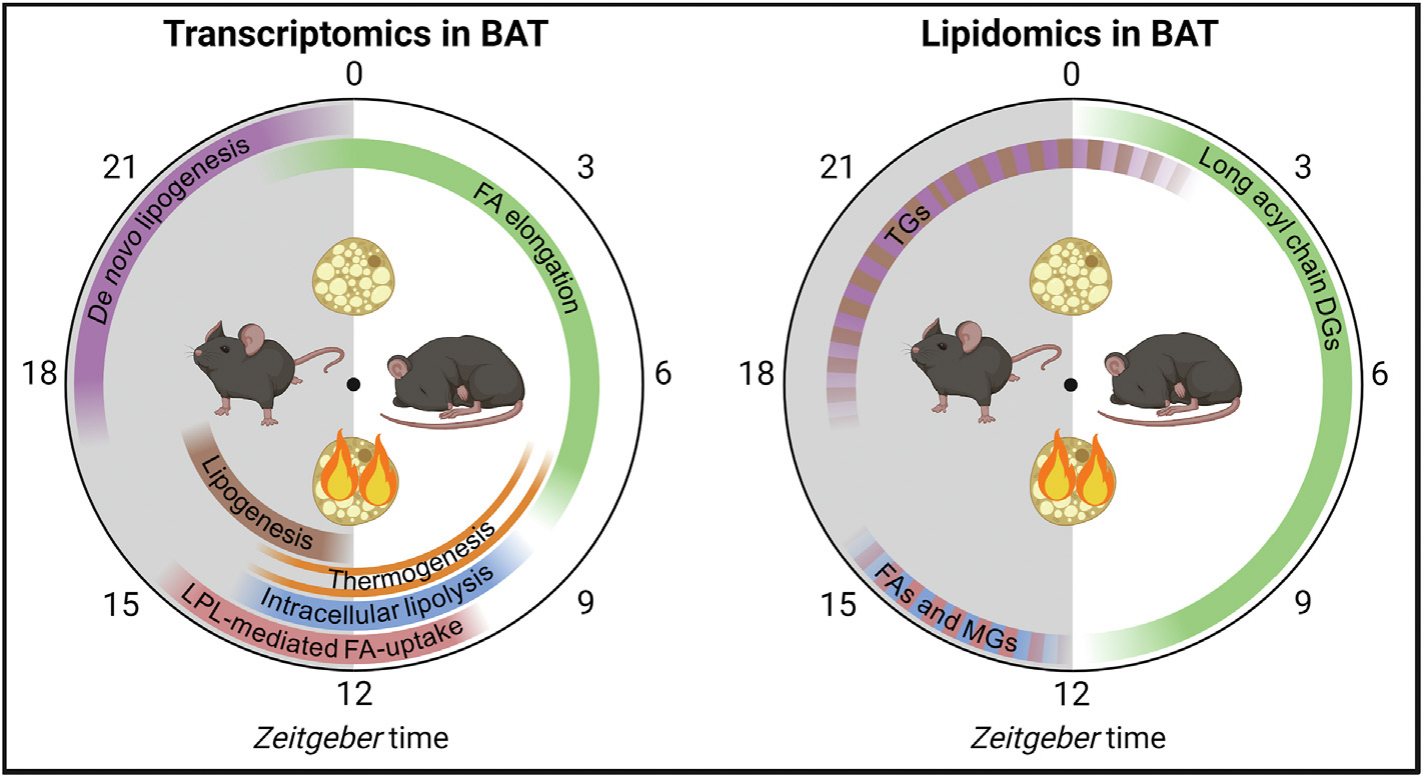
**Hypothetical model of diurnal processes in brown adipose tissue.** Around *Zeitgeber* Time (ZT) 0, the onset of the light (inactive) phase, uncoupling protein 1 (*Ucp1*) expression, which encodes a protein that uncouples ATP production from mitochondrial oxidative phosphorylation (*i.e.* thermogenesis), and thereby likely brown adipose tissue (BAT) thermogenic activity is lowest. Genes involved in fatty acid (FA) elongation peak throughout the light phase, coinciding with increased abundance of long-acyl chain diacylglycerols (DGs). In the second half of the light phase, expression of mitochondrial complexes, as well as *Ucp1* peak, suggesting peak thermogenic activity within the tissue. Genes involved in intracellular lipolysis peak shortly before the highest abundance of FAs and monoacylglycerols (MGs), possibly to supply mitochondria with FAs for β-oxidation and to allosterically activate UCP1 [1]. To replenish intracellular lipid stores, the peak in triglyceride (TG)-rich lipoprotein-derived FA-uptake follows at the onset of the dark (active) phase (*i.e.* ZT12) [13], driven by lipoprotein lipase (LPL)-mediated hydrolysis, and likely contributing to the FA and MG abundance at the onset of the dark phase. This is followed by peak expression of lipogenic genes probably to store these FAs as TGs in intracellular lipid droplets. Throughout the dark phase there is ample glucose availability from food intake, allowing for the conversion of glucose to FA during peak expression of *de novo* lipogenic genes. This may be an additional pathway to supply BAT with lipids to replenish intracellular lipid stores, resulting in highest TG abundance at the end of the dark phase.

We aimed to gain further insight into the diurnal regulation of metabolic BAT activity; the main findings of the transcriptomics and lipidomics data and our interpretations are summarized as a hypothetical model in Figure 6. Briefly, metabolic BAT activity peaks around the onset of the dark (active) phase (*i.e.* ZT12) [[9], [10], [11], [12], [13], [14], [15]]. At this time, FA for combustion are supplied through lipolysis of intracellular lipid stores and by LPL-mediated lipolysis of circulating TRLs, both possibly under transcriptional control of PPARγ. This is followed by (*de novo*) lipogenesis and FA elongation, likely to replenish intracellular lipid stores and efficient combustion during the dark phase. The relative contribution of the various processes is probably context dependent, as cold exposure adds an extra dimension to the diurnal regulation of BAT activity by, for example, increasing the amplitude and expression of genes involved in *de novo* lipogenesis during the second half of the dark phase [18], when nutrient availability is highest due to feeding. A limitation of the current study is that the experiment was performed only in male mice that were housed at room temperature.

The question remains what the main driving force is of the diurnal metabolic activity of BAT. Previous experiments indicated that oscillating BAT activity is modulated by glucocorticoids [15], but is independent of glucocorticoid receptor expression in BAT, suggestive of an indirect mechanism. Sympathetic denervation of BAT resulted in attenuated oscillations of TG-derived FA-uptake by BAT, but those experiments should be interpreted with caution given the complete abolishment of metabolic activity [13,44]. Based on the current study we suggest that stimulation of intracellular lipolysis [1] around wakening, possibly as a result of increased sympathetic activity, promotes thermogenesis and activation of PPARγ [45] to regulate expression of genes involved in uptake, storage [46], and intracellular lipolysis of lipids. These data may explain why circadian disruption by prolonged daily light exposure [44] or by flattened corticosterone oscillation [15] attenuates TG-derived FA-uptake by BAT and promotes adiposity in mice.

The current study primarily focused on BAT because it shows a strong diurnal rhythm in TG-derived FA-uptake as opposed to *e.g.* WAT [13]. However, we anticipate that a comparable approach in other tissues may provide valuable insights in the mechanisms driving their diurnal oscillations and contribute to the development of novel pharmacological strategies. Here, we identified oscillations of ANGPTL4 and LPL as important mediators in the diurnal regulation of metabolic BAT activity. ANGPTL4 is considered a therapeutic target for reducing cardiometabolic disease, as in humans, loss-of-function gene variants are associated with reduced circulating TG levels and lower cardiovascular disease risk, which could be mimicked in mice with the use of anti-ANGPTL4 monoclonal antibodies [[47], [48], [49], [50], [51], [52], [53]]. However, our data suggest that the ability of ANGPTL4 to modulate LPL activity might be dependent on time of day. ANGTPL4 inhibition at the onset of the resting phase may therefore be clinically most relevant for stimulating TG-derived FA-uptake by BAT.

## 4 Materials and methods

### 4.1 Animals

All mice were housed under standard conditions with a 12h:12h light:dark schedule at 22 °C with *ad libitum* access to a chow diet (Rat and Mouse No.3 Breeding, SDS, Horley, United Kingdom) and water. All mouse experiments were conducted in accordance with the Institute for Laboratory Animal Research Guide for the Care and Use of Laboratory Animals and were approved by the National Committee for Animal experiments.

In a first experiment, non-fasted male C57BL/6J mice (Charles River Laboratories, Wilmington, MA, USA; 10 weeks old) were killed by CO_2_ inhalation at eight time points over a 24-hour period (corresponding to ZT0, ZT3, ZT6, ZT9, ZT12, ZT15, ZT18 and ZT21; *n* = 4 per time point). iBAT was collected and snap-frozen to assess oscillating gene expression by RNA-sequencing, oscillating lipid levels by lipidomics, and oscillating chromatin binding of PPARγ by ChIP-sequencing (see below). In a second and third experiment, male whole-body *Angptl4* KO and *Angptl4* Tg mice, respectively, were compared with C57BL/6J mice (both on a C57BL/6J background; in-house breeding), which were obtained as described previously [54,55]. *Angptl4* Tg mice were compared to littermates and *Angptl4* KO mice to mice from another in-house breeding. *Angptl4* KO mice do not fully express the *Angptl4* gene, resulting in a non-functional ANGPTL4 protein [54,55], whereas *Angptl4* Tg mice overexpress *Angptl4* under its own promotor [56]. *Angptl4* KO (9–12 weeks old; *n* = 7–9 per group per time point), *Angptl4* Tg (9–12 weeks old; *n* = 8 per group per time point) and their WT controls were killed at the onset of the light phase (corresponding to ZT0) and at the onset of the dark phase (corresponding to ZT12) to assess oscillating organ uptake of TG-derived FA (see below).

### 4.2 RNA sequencing

Total RNA of iBAT (approx. 10–20 mg; experiment 1) was isolated using the nucleospin kit (Macherey–Nagel, Düren, Germany; 740955.50) after homogenization by a FastPrep-24™ 5G bead beating grinder and lysis system (4.0 m s^−1^, 10 s; MP Biomedicals™, Santa Ana, California, USA) in a mixture of 400 μL RA1 (Macherey–Nagel, Düren, Germany; 740955.50) with 4 μL β-mercaptoethanol in Lysing Matrix D tubes (MP Biomedicals™, Santa Ana, California, USA; 116913500). RNA was quality-controlled on a 2100 Bioanalyzer (Agilent Technologies, Santa Clara, California, USA).

Sequencing quality was assessed with MultiQC [57], reads were mapped to the *Mus Musculus* reference genome mm10 (Ensembl build 38.88) using GSNAP (version 2017-09-11) [58] and reads per gene were counted using HTSeq-count (version 0.6.1) [59] and the Ensembl gene annotation version 38.88. Genes were filtered by expression and normalized using edgeR (version 3.30.3) [60]. Oscillation of gene expression was assessed by JTK cycle [61] (MetaCycle version 1.2.0). Heatmaps of standardized residuals were generated using the ‘heatmap.2’ function using the complete linkage method and the complement of the Pearson distance (gplots version 3.0.4). Functional enrichment by gene ontology was performed using GOrilla [62], and transcription factor enrichment analyses were performed using ChIP-X enrichment analysis version 3 (ChEA3) [63].

### 4.3 ChIP-sequencing

ChIP was performed as previously described [64]. Briefly, iBAT samples pooled from eight mice (approx. 45 mg per mouse combined; experiment 1) were homogenized in lysis buffer (10 mM HEPES-KOH, pH 7.3, 10 mM KCl, 5 mM MgCl_2_, 0.5 mM DTT, 1 × cOmplete™ Protease Inhibitor (Roche Diagnostics, Almere, The Netherlands)), filtered through a Falcon® 70 μm cell strainer (Life Sciences, Corning, New York, USA), cross-linked in 10 mL 1% formaldehyde in PBS for 15 min and quenched with 1.5 mL 1 M glycine for 5 min. After washing, pelleted nuclei were further lysed twice by resuspension in 1 mL cold fast IP buffer (50 mM NaCl, 50 mM Tris–HCl (pH 7.5), 5 mM EDTA, 0.5% v/v NP40, 1% v/v Triton X-100, 1 × cOmplete™ Protease Inhibitor) and passing through a 24G syringe. Chromatin was subsequently sheared (Bioruptor® plus, Diagenode, Liège, Belgium) in 1 mL shearing buffer (1% v/v SDS, 10 mM EDTA, 50 mM Tris–HCl (pH 8), 1 × cOmplete™ Protease Inhibitor). Sheared chromatin was then centrifuged, and 10% of each sample was stored at −20 °C to be used as input. The chromatin was diluted in 7.2 mL dilution buffer (0.01% v/v SDS, 1.1% v/v Triton X-100, 1.2 mM EDTA, 16.7 mM Tris–HCl (pH 8.0), 0.167 M NaCl, 1 × cOmplete™ Protease Inhibitor) and incubated overnight at 4 °C with 8 μg of PPARγ antibody (2443, Cell Signaling Technology, Danvers, Massachusetts, USA). Chromatin was cleared by centrifugation, and the top 90% was incubated with 100 μL bovine serum albumin-blocked DynaBeads™ (11203D; Thermo Fisher Scientific, Waltham, USA) for 6 h at 4 °C. Unbound complexes were removed by washing with fast IP buffer. Beads were collected and washed in Tris–EDTA buffer (10 mM Tris–HCl, 1 mM disodium EDTA, pH 8.0), and immunoprecipitated chromatin was collected by elution with 100 μL beads elution buffer (0.105 M NaHCO_3_, 1% SDS) for 15 min at room temperature two times. Both the input sample and the immunoprecipitated chromatin were de-cross-linked by addition of 4 μL and 8 μL 5 M NaCl, respectively, followed by overnight incubation at 65 °C. Samples were subsequently treated with RNAse A and Proteinase K, and DNA was purified using MinElute® columns from the PCR Purification Kit (QIAGEN, Hilden, Germany) using the manufacturer's protocol.

Sequencing quality was assessed with MultiQC [57], reads were mapped to the *Mus Musculus* reference genome mm10 (Ensembl build 38.88) using BWA-MEM (version 0.7.17) [65] and peaks were called using MACS2 (version 2.1.2) [66]. Reads per peak were counted using HTSeq-count (version 1.99.2) [59]. Peak annotation was performed using HOMER (version 4.11) [67] with the mouse reference dataset mm10.v6.4.zip. Peaks that were significant (FDR<0.05) in at least six out of eight samples were filtered by expression and normalized using edgeR (version 3.30.3) [60]. Oscillation of binding sites was assessed by JTK cycle [61] (MetaCycle version 1.2.0).

### 4.4 Lipidomics

Lipidomics was performed as previously described [34,68], with minor adjustments. Briefly, the following amounts of internal standards dissolved in 1:1 (v/v) methanol:chloroform were added to each sample: bis(monoacylglycero)phosphate BMP(14:0)2 (0.2 nmol), ceramide-1-phosphate C1P (d18:1/12:0) (0.127 nmol), D_7_-cholesteryl ester CE(16:0) (2 nmol), ceramide Cer(d18:1/12:0) (0.118 nmol), ceramide Cer(d18:1/25:0) (0.130 nmol), cardiolipin CL(14:0)4 (0.1 nmol), diacylglycerol DAG(14:0)2 (0.5 nmol), glucose ceramide GlcCer(d18:1/12:0) (0.126 nmol), lactose ceramide LacCer(d18:1/12:0) (0.129 nmol), lysophosphatidicacid LPA(14:0) (0.1 nmol), lysophosphatidylcholine LPC(14:0) (0.5 nmol), lysophosphatidylethanolamine LPE(14:0) (0.1 nmol), lysophosphatidylglycerol LPG(14:0) (0.02 nmol), phosphatidic acid PA(14:0)2 (0.5 nmol), phosphatidylcholine PC(14:0)2 (2 nmol), phosphatidylethanolamine PE(14:0)2 (0.5 nmol), phosphatidylglycerol PG(14:0)2 (0.1 nmol), phosphatidylinositol PI(8:0)2 (0.5 nmol), phosphatidylserine PS(14:0)2 (5 nmol), sphinganine 1-phosphate S1P(d17:0) (0.124 nmol), sphinganine-1-phosphate S1P(d17:1) (0.125 nmol), ceramide phosphocholines SM(d18:1/12:0) (2.129 nmol), sphingosine SPH(d17:0) (0.125 nmol), sphingosine SPH(d17:1) (0.125 nmol), triacylglycerol TAG(14:0)2 (0.5 nmol). 1.5 mL 1:1 (v/v) methanol:chloroform was added before thorough mixing. Each sample was then centrifuged (10 min; 14,000 rpm), supernatant was evaporated under a stream of nitrogen at 60 °C and reconstituted in 150 μL of 1:1 (v/v) methanol:chloroform. Lipids were analyzed using a Thermo Scientific Ultimate 3000 binary HPLC coupled to a Q Exactive Plus Orbitrap mass spectrometer. For normal phase separation, 2 μL of each sample was injected onto a Phenomenex® LUNA silica, 250 × 2 mm, 5 μm 100 Å. Column temperature was held at 25 °C. Mobile phase consisted of (A) 85:15 (v/v) methanol:water containing 0.0125% formic acid and 3.35 mmol/L ammonia and (B) 97:3 (v/v) chloroform:methanol containing 0.0125% formic acid. Using a flow rate of 0.3 mL/min, the LC gradient consisted of: 10% A for 0–1 min, reach 20% A at 4 min, reach 85% A at 12 min, reach 100% A at 12.1 min, 100% A for 12.1–14 min, reach 10% A at 14.1 min, 10% A for 14.1–15 min. For reversed phase separation, 5 μL of each sample was injected onto a Waters HSS T3 column (150 × 2.1 mm, 1.8 μm particle size). Column temperature was held at 60 °C. Mobile phase consisted of (A) 4:6 (v/v) methanol:water and B 1:9 (v/v) methanol:isopropanol, both containing 0.1% formic acid and 10 mmol/L ammonia. Using a flow rate of 0.4 mL/min, the LC gradient consisted of: 100% A at 0 min, reach 80% A at 1 min, reach 0% A at 16 min, 0% A for 16–20 min, reach 100% A at 20.1 min, 100% A for 20.1–21 min. MS data were acquired using negative and positive ionization using continuous scanning over the range of *m*/*z* 150 to *m*/*z* 2000. Data were analyzed using an in-house developed metabolomics pipeline written in the R programming language (http://www.r-project.org/). All reported lipids were normalized to corresponding internal standards according to lipid class. Lipid identification has been based on a combination of accurate mass, (relative) retention times, fragmentation spectra and the injection of relevant standards. Oscillation of lipid species was assessed by JTK cycle [61] (MetaCycle version 1.2.0). Heatmaps of standardized residuals were generated using the ‘heatmap.2’ function using the complete linkage method and the complement of the Pearson distance (gplots version 3.0.4).

### 4.5 TG-derived FA-uptake by organs

Prior to killing, mice of experiment 2 and 3 were fasted for 4 h and received an intravenous injection of TRL-like particles (80 nm) radiolabeled with glycerol tri[^3^H]oleate, prepared as described previously [69] (1 mg TG in 200 μL saline per mouse). After 15 min, mice were killed by CO_2_ inhalation, perfused via the heart with ice-cold PBS and iBAT and subscapular BAT (sBAT) were collected and weighed. Half of the tissues (approx. 30–35 and 15–20 mg for iBAT and sBAT, respectively) were dissolved overnight at 56 °C in 0.5 mL Solvable (PerkinElmer, Waltham, Massachusetts, USA) and diluted in 5 mL Ultima Gold (PerkinElmer, Waltham, Massachusetts, USA). ^3^H-activity was measured in a liquid scintillation counter (Tri-Carb 2910 TR, PerkinElmer, Waltham, Massachusetts, USA) and expressed as a percentage of injected dose per whole organ.

### 4.6 iBAT LPL protein quantification

Frozen iBAT samples (approx. 10 mg) (experiment 2 and 3) were lysed in RIPA buffer (150 mM sodium chloride, 1.0% Triton X-100, 0.5% sodium deoxycholate, 0.1% sodium dodecyl sulphate, 50 mM Tris pH 8.0, protease & phosphatase inhibitors (Thermo Fisher Scientific, Waltham, USA)), homogenized by a FastPrep-24™ 5G bead beating grinder and lysis system (4.0 m · s^−1^, 10 s; MP Biomedicals™, Santa Ana, California, USA) and repeatedly centrifuged (5 min; 16.2 · *g*; 4 °C) to remove fat. Protein concentrations were determined using the Pierce™ BCA Protein Assay Kit (Thermo Fisher Scientific, Waltham, USA), according to manufacturer's protocol. LPL abundance was analyzed by Western blot using a goat anti-mouse LPL antibody (1:1000; kind gift from André Bensadoun), for which 20 μg protein was loaded. Rabbit anti-mouse glyceraldehyde 3-phosphate dehydrogenase (GAPDH) antibody (1:1000; sc-25778, Santa Cruz, Dallas, Texas, USA) was used for normalization. Anti-goat (1:5000) and anti-rabbit (1:1000) antibodies were used for LPL and GAPDH, respectively. Relative normalized protein levels were quantified by Image Lab software (Bio-Rad).

### 4.7 iBAT LPL activity assay

iBAT samples (approx. 10 mg) (experiment 2 and 3) were manually cut with a razor blade in order to keep cells intact, and dissolved in DMEM (31966, ThermoFisher Scientific, Waltham, Massachusetts, USA) containing bovine serum albumin (0.5%) and heparin (0.0004%) to allow for discrimination between extracellular LPL and intracellular LPL. Samples were incubated for 1 h at 37 °C and centrifuged (10 min; 16.2 · *g*; 4 °C), and the middle layer containing heparin-bound LPL was isolated and used for *in vitro* assessment of LPL activity. Briefly, 100 μL tissue extract was added to 200 μL substrate solution (9.2 mg/mL triolein (T7-140, Sigma, Saint Louis, Missouri, USA), 2.5 μCI/mL glycerol-tri-(9,10–^3^H)-oleate (NET431L005MC, PerkinElmer, Waltham, Massachusetts, USA), 0.1% Triton X-100, 0.1 M Tris.HCl pH 8.6 (1.08382.100, Merck, Burlington, Massachusetts, USA), 1% free fatty acid-free bovine serum albumin (A6003, Sigma, Saint Louis, Missouri, USA), and 20% human serum. After 60 and 120 min, a mix of heptane: methanol: chloroform (in a ratio of 1 : 1.28: 1.37 v/v) and K_2_CO_3_ (0.1 M) was added to stop the reaction, samples were diluted in 2.5 mL Ultima Gold (PerkinElmer, Waltham, Massachusetts, USA), and ^3^H-activity was measured in a liquid scintillation counter (Tri-Carb 2910 TR, PerkinElmer, Waltham, Massachusetts, USA) to calculate TG hydrolase activity.

### 4.8 sBAT gene expression analysis

Total RNA was isolated from frozen sBAT (approx. 10 mg) (experiment 2 and 3) through lysing in TriPure RNA Isolation Reagent (Roche Diagnostics, Almere, The Netherlands) and homogenization by a FastPrep-24™ 5G bead beating grinder and lysis system (4.0 m · s^−1^, 10 s; MP Biomedicals™, Santa Ana, California, USA). Subsequently, 1 μg cDNA was synthesized using Moloney Murine Leukemia Virus Reverse Transcriptase (M-MLV RT; Promega, Madison, Wisconsin, USA) according to the manufacturer's protocol. qPCR was carried out using SYBR green kit (Promega, Madison, Wisconsin, USA) on a CFX96 PCR machine (Bio-Rad, Hercules, California, USA). *Lpl* (forward primer (FW): CCCTAAGGACCCCTGAAGAC; reverse primer (RV): GGCCCGATACAACCAGTCTA) and *Angptl4* (FW: GGAAAGAGGCTTCCCAAGAT; RV: TCCCAGGACTGGTTGAAGTC) expression was normalized to the geometric mean Ct value of *Gapdh* (FW: GGGGCTGGCATTGCTCTCAA; RV: TTGCTCAGTGTCCTTGCTGGGG), *ß-tubulin* (FW: CCGGGGCAGCCAACAGTACC; RV: CTCGGGGCGGGATGTCACAC), *ß-actin* (FW: AACCGTGAAAAGATGACCCAGAT; RV: CACAGCCTGGATGGCTACGTA), and 60S acidic ribosomal protein P0 (*Rplp0*, FW: GGACCCGAGAAGACCTCCTT; RV: GCACATCACTCAGAATTTCAATGG), and expressed relative to the WT ZT0 group using the 2^−ΔΔCT^ method.

### 4.9 Statistical analyses

*P* < 0.05 was considered statistically significant. In experiment 2 and 3, data were tested for normality by an Anderson-Darling test. Comparisons between groups were made by two-way ANOVA with a post-hoc Tukey test when data were normally distributed. In case of non-normal distributions, comparisons were made by a Kruskall–Wallis test. Statistical analyses were performed with GraphPad Prism software, version 8.4.2 (GraphPad, La Jolla, California) and R (http://www.r-project.org/; version 4.0.2). Data are presented as means ± SEM.

## Data availability

The RNA-sequencing and ChIP-sequencing datasets discussed in the current study have been deposited in NCBI's Gene Expression Omnibus (GEO) [70] and are accessible through GEO Series accession numbers GSE182045 (https://www.ncbi.nlm.nih.gov/geo/query/acc.cgi?acc=GSE182045) and GSE197261 (https://www.ncbi.nlm.nih.gov/geo/query/acc.cgi?acc=GSE197261). The lipidomics dataset discussed in the current study has been deposited in MetaboLights [71] and is accessible through study identifier MTBLS4082 (https://www.ebi.ac.uk/metabolights/MTBLS4082). The remaining datasets used and/or analyzed during the current study are available from the corresponding author on reasonable request.

## Author contributions

Conceptualization, R.V.E., W.I.H.P., M.S., S.KE., P.C.N.R., and S.KO.; Formal Analysis, R.V.E., W.I.H.P., J.B.V.K., and T.D.; Investigation, R.V.E., W.I.H.P., M.S., C.J., R.S., T.C.M.S., A.C.M.P., R.H.P.V., H.M., J.B.V.K., M.V.W., N.H.U., and S.KO.; Writing – Original Draft, R.V.E. and W.I.H.P.; Writing – Review & Editing, M.S., S.KE., P.C.N.R., and S.KO.; Supervision, S.KO.; Funding acquisition, P.C.N.R. and S.KO. Both R.V.E. and W.I.H.P. contributed equally and have the right to list their name first in their CV.

## Funding

This work is supported by the Netherlands Cardiovascular Research Initiative: an initiative with support of the 10.13039/100002129Dutch Heart Foundation (CVON2014-02 ENERGISE to P.C.N.R. and S.KE and CVON2017-20-GENIUS-II to P.C.N.R. and a CVON-GENIUS-II talent grant to R.V.E.), Dutch Federation of University Medical Centers, the 10.13039/501100001826Netherlands Organisation for Health Research and Development, and the Royal Netherlands Academy of Sciences. M.S. is supported by the 10.13039/501100009708Novo Nordisk Foundation (NNF18OC0032394). S.KO is supported by the 10.13039/100002129Dutch Heart Foundation (2017T016). C.J. and N.H.U. are supported by the 10.13039/501100001659Deutsche Forschungsgemeinschaft (CRC1064 chromatin dynamics and Trans-Regio TRR333 BATenergy).

## Appendix A Supplementary data

Supplemental Data Supplemental Data

RNA-sequencing RNA-sequencing

ChIP-sequencing ChIP-sequencing

Lipidomics Lipidomics
