# Supplementary material for: Angiopoietin-like 4 governs diurnal lipoprotein lipase activity in brown adipose tissue
Source: Mol Metab. 2022 Apr 10;60:101497. doi: 10.1016/j.molmet.2022.101497 (PMC9048098; doi:10.1016/j.molmet.2022.101497)
Supplement: Supplemental Data [file mmc1.pdf]

Supplemental information

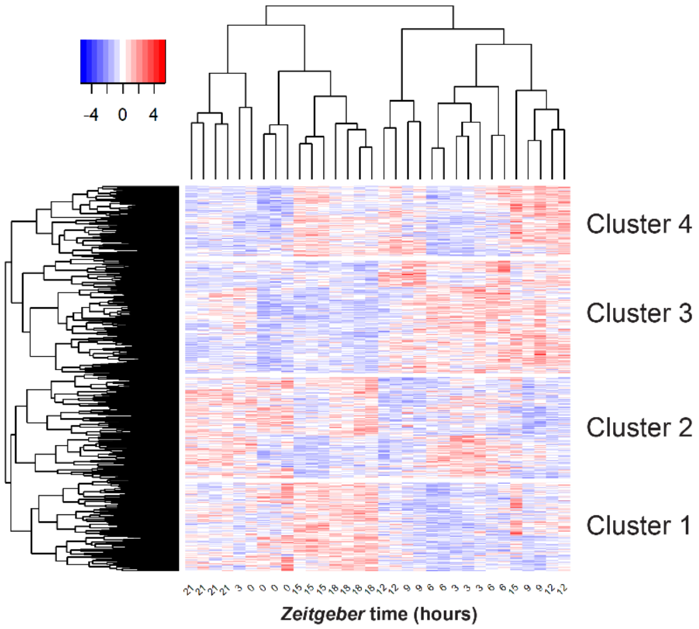

Fig S1. **The transcriptome of murine brown adipose tissue consists of four clusters with distinct oscillating expression phases**. Interscapular brown adipose tissue samples were collected from chow-fed male C57BL/6J mice at 3-hour intervals throughout a 24-hour period to produce eight time points in total (*n*=4 per time point), which were used to perform RNA-sequencing. Oscillation was assessed by JTK, and hierarchical clustering of standardized residuals (Z-scores) of all oscillating genes (*P*<0.05) was visualized in a heat map.

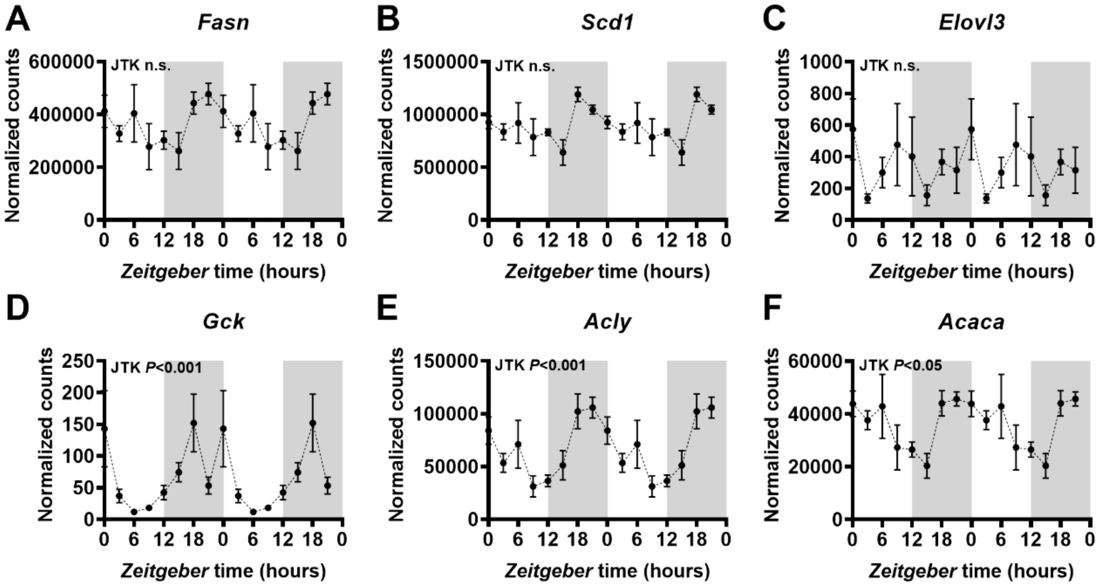

Fig S2. **Genes involved in *de novo* lipogenesis show diurnal oscillations with peak expression in the second half of the dark phase.** Interscapular brown adipose tissue samples were collected from chow-fed male C57BL/6J mice at 3-hour intervals throughout a 24-hour period to produce eight time points in total (*n*=4 per time point), which were used to perform RNA-sequencing. Oscillation was assessed by JTK, and normalized counts of **(A)** fatty acid synthase (*Fasn*), **(B)** stearoyl-CoA desaturase-1 (*Scd1*) **(C)** elongation of very long chain fatty acids protein 3 (*Elovl3*), **(D)** glucokinase (*Gck*), **(E)** ATP citrate lyase (*Acly*), and **(F)** acetyl-CoA carboxylase alpha (*Acaca*) were double plotted.

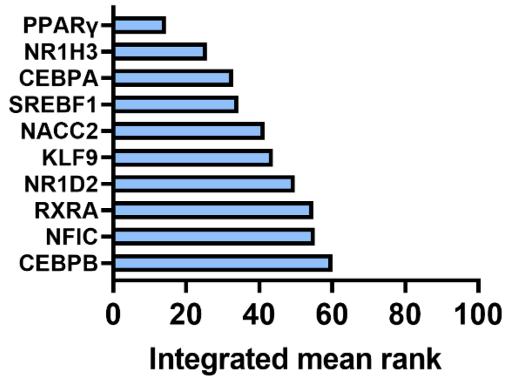

Fig S3. **Transcription factor enrichment analysis on the top 100 genes with the largest oscillation amplitude identified PPARγ as the top hit.** Interscapular brown adipose tissue samples were collected from chow-fed male C57BL/6J mice at 3-hour intervals throughout a 24-hour period to produce eight time points in total (*n*=4 per time point), which were used to perform RNA-sequencing. Oscillation was assessed by JTK, and enrichment by transcription factor of the 100 genes with largest absolute oscillation amplitude was performed, top 10 hits are displayed here. CEBPA, CCAAT enhancer binding protein alpha; CEBPB, CCAAT enhancer binding protein beta; KLF9, Kruppel like factor 9; NACC2, NACC family member 2; NFIC, nuclear factor I C; NR1D2, nuclear receptor subfamily 1 group D member 2; NR1H3, nuclear receptor subfamily 1 group H member 3; PPARγ, peroxisome proliferator activated receptor gamma; RXRA, retinoid X receptor alpha; SREBF1, sterol regulatory element binding transcription factor 1.

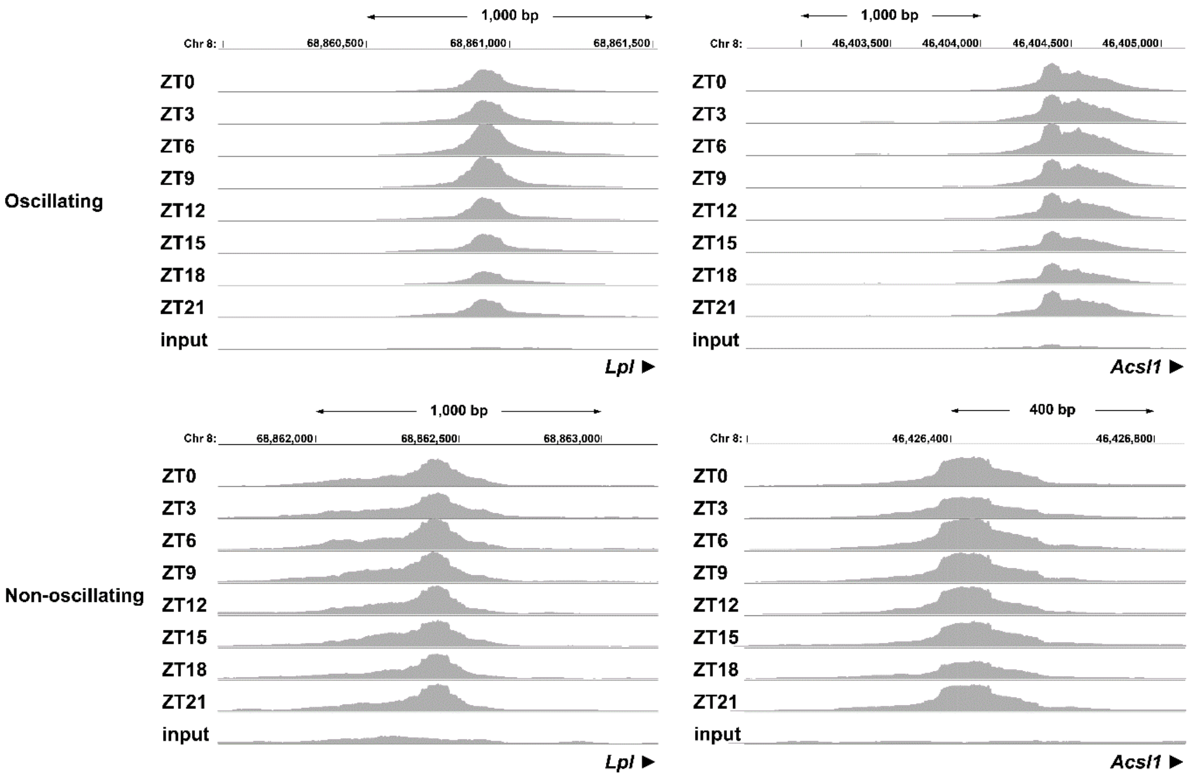

Fig S4. **Representative PPARγ ChIP-sequencing tracks**. Interscapular brown adipose tissue samples were collected from chow-fed male C57BL/6J mice at 3-hour intervals throughout a 24-hour period to produce eight time points in total (*n*=8 per time point). On pooled samples, chromatin immunoprecipitation (ChIP)-sequencing was performed for PPARγ. Oscillation was assessed by JTK, and representative ChIP-sequencing tracks of oscillating (*P*<0.05) and non-oscillating peaks annotated to lipoprotein lipase (*Lpl*) or acyl-CoA synthetase long chain family member 1 (*Acsl1*) were visualized.

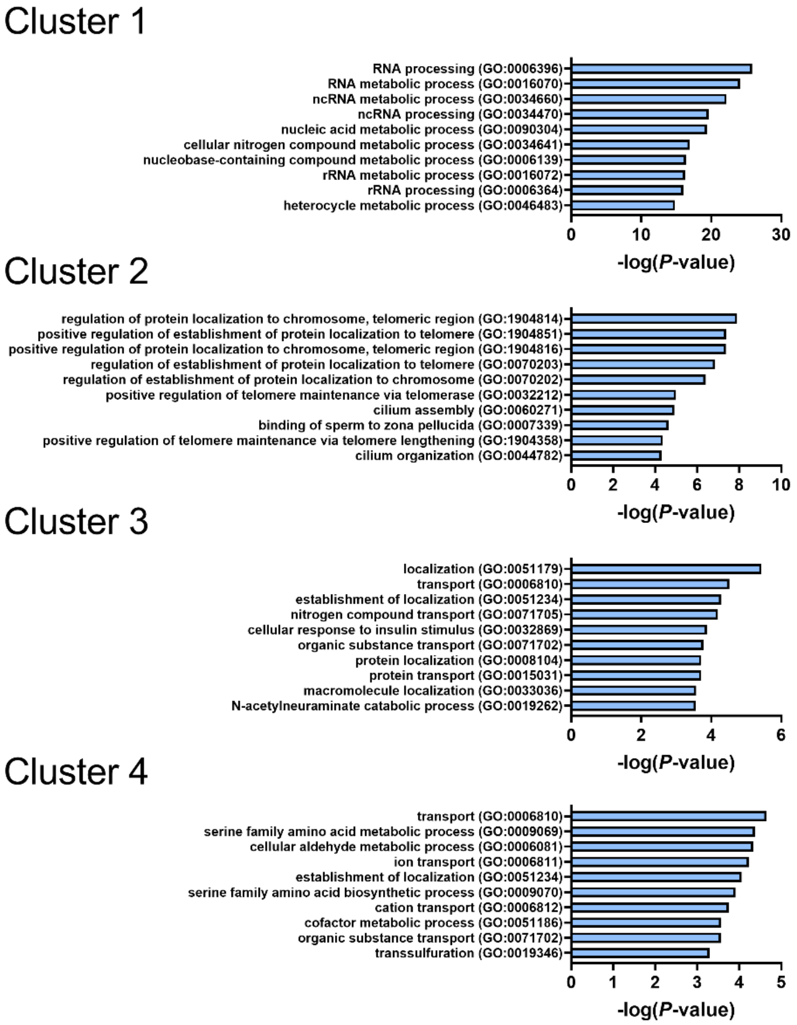

Fig S5. **Gene ontology enrichment analysis for oscillating genes with non-oscillating PPARγ binding**. Interscapular brown adipose tissue samples were collected from chow-fed male C57BL/6J mice at 3-hour intervals throughout a 24-hour period to produce eight time points in total (*n*=8 per time point). On pooled samples, chromatin immunoprecipitation (ChIP)-sequencing was performed for PPARγ, and oscillation of peaks was assessed by JTK. Peaks were annotated, and within each of the four gene clusters with distinct expression phases as identified by RNA-sequencing (Fig 1), functional enrichment by gene ontology was performed on the genes with non-oscillating ($P \geq 0.05$) PPARγ binding, and top 10 hits are depicted.

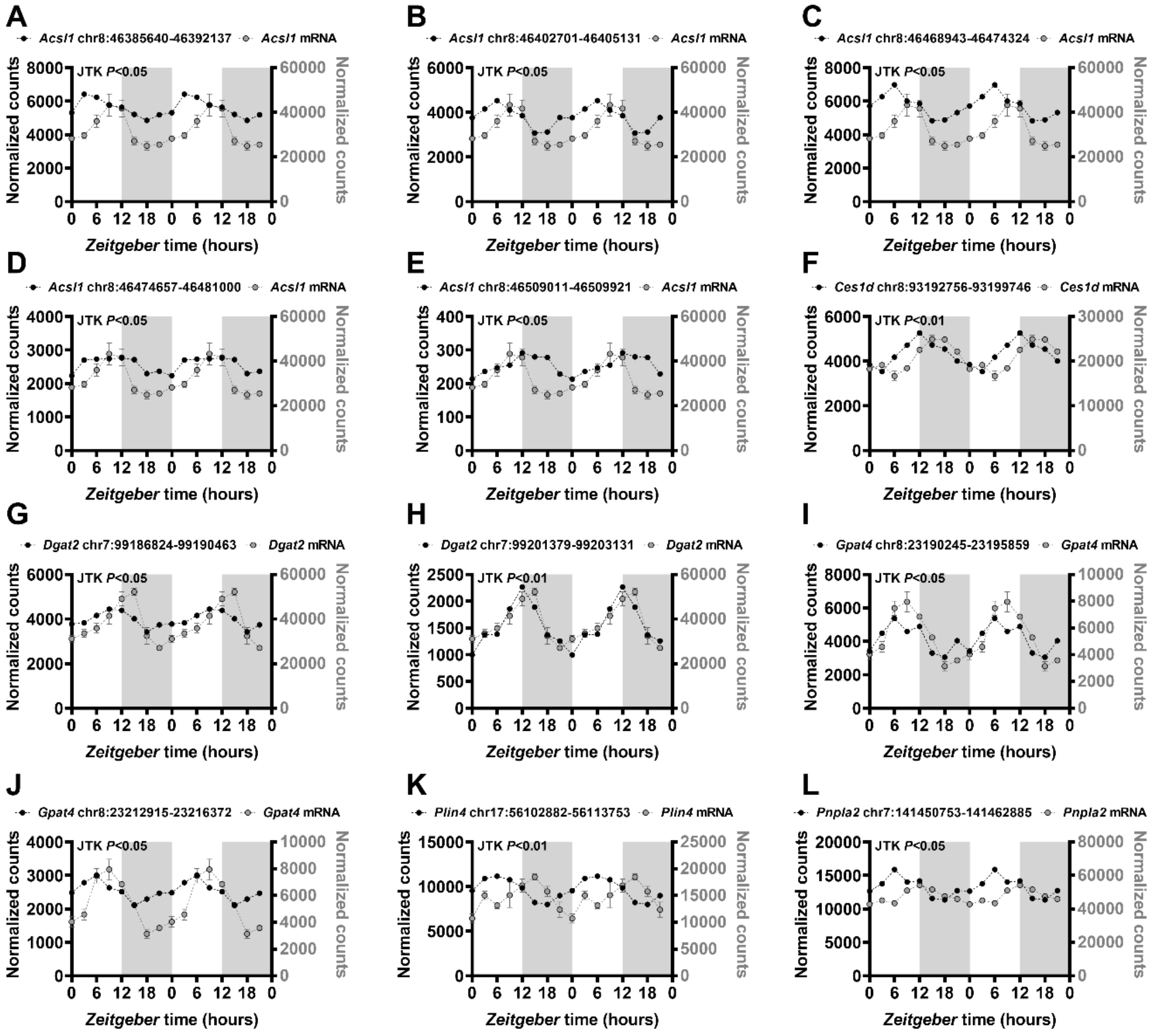

Fig S6. **Genes with largest diurnal amplitude that are involved in lipid storage and intracellular lipolysis show oscillating PPARγ binding**. Interscapular brown adipose tissue samples were collected from chow-fed male C57BL/6J mice at 3-hour intervals throughout a 24-hour period to produce eight time points in total (*n*=8 per time point). On pooled samples, chromatin immunoprecipitation (ChIP)-sequencing was performed for PPARγ. Oscillation was assessed by JTK, and normalized counts of oscillating (*P*<0.05) peaks annotated to **(A-E)** acyl-CoA synthetase long chain family member 1 (*Acsl1*), **(F)** carboxylesterase 1 D (*Ces1d*) **(G-H)** diacylglycerol O-acyltransferase 2 (*Dgat2*) **(I-J)** glycerol-3-phosphate acyltransferase 4 (*Gpat4*), **(K)** perilipin 4 (*Plin4*) and **(L)** patatin like phospholipase domain containing 2 (*Pnpla2*) were double plotted (black lines). RNA-sequencing was performed in the same samples (*n*=4 per time point), and normalized gene counts were double plotted in the same panels (gray lines).

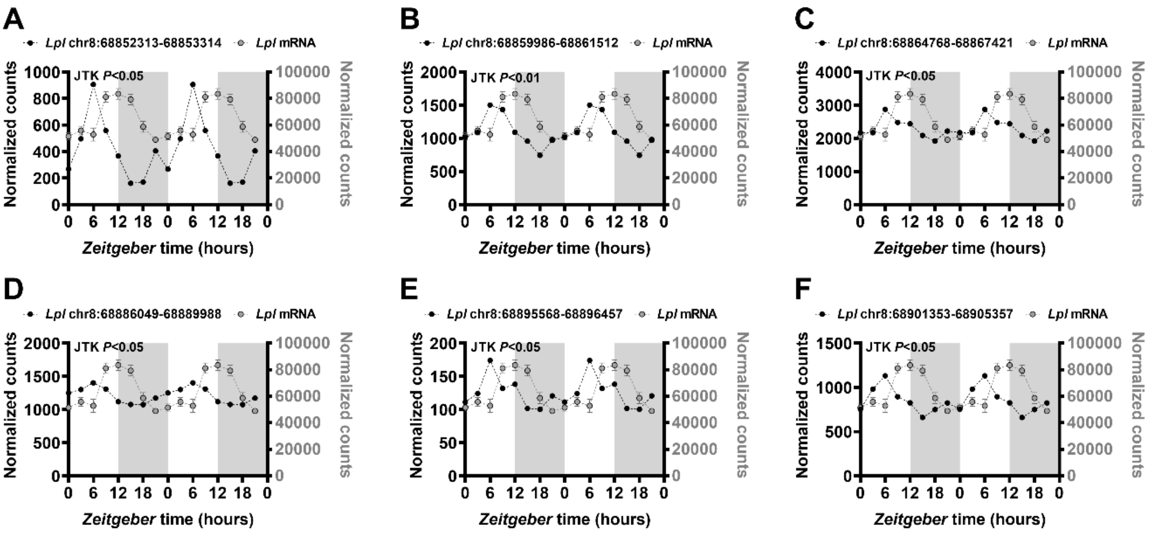

Fig S7. ***Lpl* shows oscillating PPARγ binding just prior to peak mRNA expression**. Interscapular brown adipose tissue samples were collected from chow-fed male C57BL/6J mice at 3-hour intervals throughout a 24-hour period to produce eight time points in total (*n*=8 per time point). On pooled samples, chromatin immunoprecipitation (ChIP)-sequencing was performed for PPARγ. Oscillation was assessed by JTK, and normalized counts of oscillating (*P*<0.05) peaks annotated to **(A-F)** lipoprotein lipase (*Lpl*) were double plotted (black lines). RNA-sequencing was performed in the same samples (*n*=4 per time point), and normalized gene counts were double plotted in the same panels (gray lines).

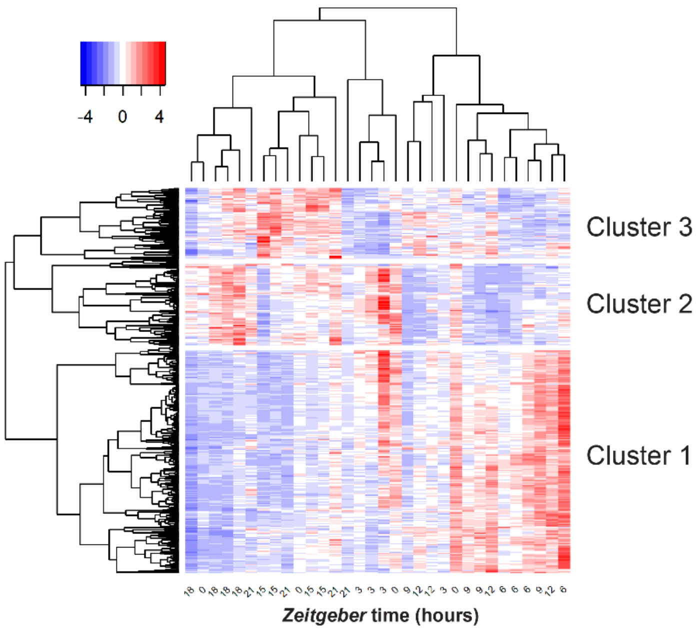

Fig S8. **The lipidome of murine brown adipose tissue consists of three clusters with distinct oscillating phases**. Interscapular brown adipose tissue samples were collected from chow-fed male C57BL/6J mice at 3-hour intervals throughout a 24-hour period to produce eight time points in total (*n*=4 per time point), which were used to perform ultra-performance liquid chromatography (UPLC)-high-resolution mass spectrometry (HRMS)-based lipidomics. Oscillation was assessed by JTK, and hierarchical clustering of standardized residuals (Z-scores) of oscillating (*P*<0.05) lipid species was visualized in a heat map.

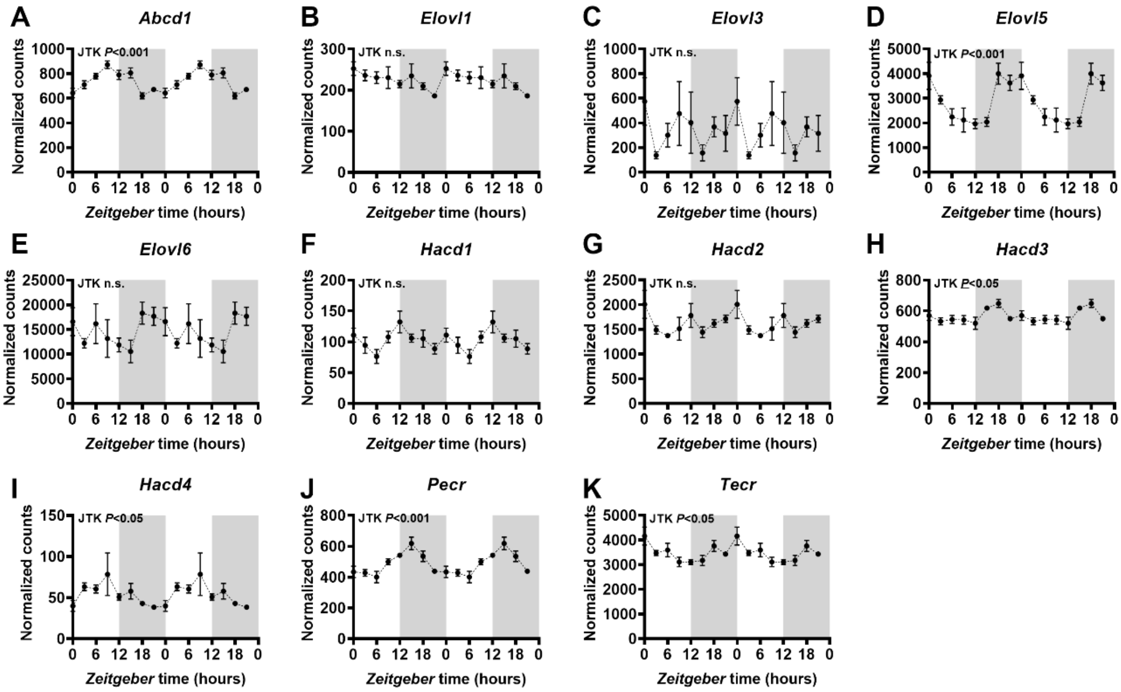

Fig S9. **Genes involved in fatty acid elongation show diurnal oscillations with peak expression around the onset of the light phase.** Interscapular brown adipose tissue samples were collected from chow-fed male C57BL/6J mice at 3-hour intervals throughout a 24-hour period to produce eight time points in total (*n*=4 per time point), which were used to perform RNA-sequencing. Oscillation was assessed by JTK, and normalized counts of **(A)** ATP binding cassette subfamily D member 1 (*Abcd1*), **(B)** elongation of very long chain fatty acid protein (*Elovl*)*1*, **(C)** *Elovl3*, **(D)** *Elovl5*, **(E)** *Elovl6*, **(F)** 3-hydroxyacyl-CoA dehydratase (*Hacd*)*1*, **(G)** *Hacd2*, **(H)** *Hacd3*, **(I)** *Hacd4*, **(J)** peroxisomal trans-2-enoyl-CoA reductase (*Pecr*) and **(K)** trans-2,3-enoyl-CoA reductase (*Tecr*) were double plotted.

Table S1. **Comparison of oscillation amplitudes of individual genes within clusters 3 and 4.**

**Cluster 3**					
**Gene**	**ADJ.P**	**PER**	**LAG**	**REL.AMP**	**Function**
Nr1d1	2.40E-12	24	7.5	1.03	Core clock
B430219N15Rik	1.13E-05	21	10.5	0.73	*Long non-coding RNA*
Cntfr	9.13E-06	21	10.5	0.71	Cytokine binding
Cyp2e1	1.74E-04	21	12	0.68	P450 pathway
Ass1	2.43E-09	21	10.5	0.66	Arginine biosynthesis
Gm5424	1.60E-08	21	10.5	0.63	*Processed pseudogene*
Mt2	4.38E-07	21	7.5	0.62	Zinc and copper homeostasis
Ehhadh	5.70E-05	21	6	0.62	Peroxisomal beta-oxidation
Nr1d2	4.59E-14	21	10.5	0.62	Core clock
Klf15	1.18E-07	21	9	0.59	Transcriptional regulator
**Gene**	**ADJ.P**	**PER**	**LAG**	**AMP**	**Function**
mt-Nd1	1.5E-07	21	7.5	58155.7	Mitochondrial electron transport
mt-Nd5	1.3E-03	21	9	45832.3	Mitochondrial electron transport
mt-Cytb	1.4E-02	21	9	40922.1	Mitochondrial electron transport
mt-Nd2	1.1E-03	21	6	39281.4	Mitochondrial electron transport
Ucp1	2.8E-03	24	7.5	7927.2	Mitochondrial uncoupling of ATP production
Acsl1	2.4E-06	24	9	6460.3	Intracellular lipolysis
Atp1a2	1.7E-05	21	9	4096.7	Osmoregulation
Prkar2b	3.2E-05	21	9	3282.3	Sympathetic signaling
Pfkfb3	3.7E-03	21	6	2016.6	Stimulation of glycolysis
Gpat4	2.2E-08	24	9	1958.0	Lipogenesis

**Cluster 4**					
**Gene**	**ADJ.P**	**PER**	**LAG**	**REL.AMP**	**Function**
Dbp	4.20E-17	21	10.5	1.21	Core clock
Per2	2.53E-15	21	15	0.93	Core clock
Per3	3.75E-12	24	13.5	0.75	Core clock
Ciart	1.60E-08	21	12	0.72	Core clock transcription factor
Nnat	2.95E-04	21	12	0.68	Glycogen metabolism

Tef	1.37E-11	24	13.5	0.63	Transcription factor
Hlf	2.13E-10	24	12	0.62	Transcription factor
Gm45909	4.38E-07	21	13.5	0.61	*To be experimentally confirmed*
Usp2	8.69E-09	24	13.5	0.61	Cell cycle regulation
Lep	3.78E-06	21	16.5	0.61	Hormone

**Gene**	**ADJ.P**	**PER**	**LAG**	**AMP**	**Function**
Lpl	2.4E-06	21	12	18714.7	Extracellular lipolysis
Car3	2.1E-03	21	12	13382.4	Acid-base homeostasis
Dgat2	7.4E-06	21	12	10019.0	Lipogenesis
Glul	5.1E-08	21	15	9908.9	Glutamine biosynthesis
Pnpla2	1.5E-07	24	13.5	4870.4	Intracellular lipolysis
Cidec	7.2E-07	21	13.5	4264.1	Lipid storage
Ces1d	3.4E-07	24	18	3700.6	Intracellular lipolysis
Plin4	3.7E-03	24	13.5	2663.3	Intracellular lipolysis
Tef	1.4E-11	24	13.5	2650.3	Transcription factor
Slc1a5	3.0E-04	21	15	2260.5	Amino acid metabolism

Interscapular brown adipose tissue samples were collected from chow-fed male C57BL/6J mice at 3-hour intervals throughout a 24-hour period to produce eight time points in total (*n*=4 per time point), which were used to perform RNA-sequencing. Oscillation was assessed by JTK, and hierarchical clustering of standardized residuals (Z-scores) of all oscillating (*P*<0.05) genes was performed (Fig 1). Genes within cluster 3 and 4 were sorted by amplitude (AMP) and relative amplitude (REL.AMP), and period (PER), phase lag (LAG, *i.e.* hours from ZT0 to peak) and AMP of top 10 hits are presented. ADJ.P, adjusted *P* value; *Acsl1*, acyl-CoA synthetase long chain family member 1; *Ass1*, argininosuccinate synthase 1; *Atp1a2*, ATPase NA+/K+ transporting subunit alpha 2; *Car3*, carbonic anhydrase 3; *Ces1d*, carboxylesterase 1 D; *Ciart*, circadian associated repressor of transcription; *Cidec*, cell death inducing DFFA like effector C; *Cntfr*, ciliary neurotrophic factor receptor; *Cyp2e1*, cytochrome P450 family 2 subfamily e member 1; *Dbp*, D-box binding PAR BZIP transcription factor; *Dgat2*, diacylglycerol O-acyltransferase 2; *Ehhadh*, enoyl-CoA hydratase and 3-hydroxyacyl CoA dehydrogenase; *Glul*, glutamate-ammonia ligase; *Gpat4*, glycerol-3-phosphate acyltransferase 4; *Hlf*, HLF transcription factor, PAR BZIP family member; *Klf15*, Kruppel like factor 15; *Lep*, leptin; *Lpl*, lipoprotein lipase; *Mt2*, metallothionein 2a; *mt-Cytb*, mitochondrially encoded cytochrome B; *mt-Nd*, mitochondrially encoded NADH:ubiquinone oxidoreductase core subunit; *Nnat*, neuronatin; *Nr1d*, nuclear receptor subfamily 1 group D member; *Per*, period circadian regulator; *Pfkfb3*, 6-phosphofructo-2-kinase/fructose-2,6-biphosphatase 3; *Plin4*, perilipin 4; *Pnpla2*, patatin like phospholipase domain containing 2; *Prkar2b*, protein kinase cAMP-dependent type II regulatory subunit beta; *Slc1a5*, solute carrier family 1 member 5; *Tef*, tef transcription factor, PAR BZIP family member; *Ucp1*, uncoupling protein 1; *Usp2*, ubiquitin specific peptidase 2.
